# Dysregulation of the cytotoxic-humoral axis and expansion of regulatory T cells in anti-TIF1γ- positive dermatomyositis

**DOI:** 10.3389/fimmu.2026.1921818

**Published:** 2026-09-18

**Authors:** Xiaoguang Cui, Kaihong Ye, Hong Wang, Junqiao Feng, Yameng Wei, Yali Kang, Nan Xu, Bo Cao, Fuqian Lei, Ke Du, Kunyi Wu, Xin Yang, Tian Tian, Shanshan Wang, Wei Wei, Zhigang Cai, Xueyi Li, Ting La

**Affiliations:** 1 National-Local Joint Engineering Research Center of Biodiagnosis and Biotherapy, The Second Affiliated Hospital of Xi’an Jiaotong University, Xi’an, China; 2 Department of Rheumatology and Immunology, The Second Affiliated Hospital of Xi’an Jiaotong University, Xi’an, China; 3 Translational Research Institute of Henan Provincial People’s Hospital and People’s Hospital of Zhengzhou University, Tianjian Laboratory of Advanced Biomedical Sciences, Academy of Medical Sciences, Zhengzhou University, Zhengzhou, China; 4 Precision Medical Research Institute, The Second Affiliated Hospital of Xi’an Jiaotong University, Xi’an, China; 5 Department of Clinical Laboratory, The Second Affiliated Hospital of Xi’an Jiaotong University, Xi’an, China; 6 Core Research Laboratory, The Second Affiliated Hospital of Xi’an Jiaotong University, Xi’an, China; 7 Department of Nephrology, The Second Affiliated Hospital of Xi’an Jiaotong University, Xi’an, China; 8 Department of Rheumatology and Immunology, Tianjin Medical University General Hospital, Tianjin, China

**Keywords:** anti-TIF1γ subtype, dermatomyositis, malignant risk, single‐cell analysis, Treg

## Abstract

**Introduction:**

Dermatomyositis (DM), particularly the anti-TIF1γ‑positive subtype, is associated with a substantially elevated risk of malignancy. However, the peripheral immune landscape of this autoantibody-defined subset, especially in patients without concurrent cancer, remains incompletely understood.

**Methods:**

We performed single‑cell RNA sequencing of peripheral blood mononuclear cells (PBMCs) from five patients with anti‑TIF1γ‑positive DM (the subtype with the highest malignancy risk) and compared their profiles with those of two other DM subtypes: anti‑MDA5‑positive DM (intermediate malignancy risk) and anti‑synthetase syndrome (ASyS, low malignancy risk).

**Results:**

All three subtypes exhibited profound immune remodeling, characterized by reduced frequencies of cytotoxic lineage cells. Anti‑TIF1γ‑positive DM showed a compensatory shift toward humoral immunity, with increased B‑lineage cells, whereas classical monocyte elevation was specific to the anti‑MDA5‑positive DM and ASyS subtypes. Notably, anti‑TIF1γ‑positive DM was uniquely distinguished by an increased frequency of immunosuppressive regulatory T (Treg) cells, a finding validated by immunohistochemistry in patient skin tissue.

**Conclusion:**

These results delineate a peripheral immunological signature of anti‑TIF1γ‑positive DM, marked by compromised cytotoxic surveillance, aberrant humoral responses, and selective Treg enrichment. We hypothesize that this Treg elevation may contribute to an immunosuppressive milieu that favors malignancy, although this mechanism remains speculative and requires functional validation in prospective cohorts.

## Introduction

Dermatomyositis (DM) is an idiopathic inflammatory myopathy (IIM) characterized by a chronic autoimmune disorder that leads to progressive muscle weakness and distinct cutaneous manifestations (1). Myositis-specific antibodies (MSAs) play a critical role in the diagnostic evaluation and subclassification of IIM, as they correlate with specific clinical phenotypes (2, 3). Key MSAs associated with DM include antibodies against Mi2, melanoma differentiation-associated gene 5 (MDA5), transcriptional intermediary factor 1γ (TIF1γ), nuclear matrix protein 2 (NXP2), and small ubiquitin-like modifier activating enzyme 1/2 (SAE1/2) (4, 5). Among these, anti-TIF1γ and anti-NXP2 antibodies are both linked to an elevated risk of malignancy (3, 4), with up to 84% of anti-TIF1γ-positive patients presenting with cancer-associated DM (1, 6, 7).

The risk of malignancy among IIM subtypes has been well-defined (8). Anti-MDA5-positive DM is a distinct autoimmune subtype characterized by a high frequency of interstitial lung disease (ILD) and an intermediate risk of malignancy (8, 9). Single-cell profiling of PBMCs has highlighted the critical role of monocytes in this specific disease subset (10–12). In contrast, anti-synthetase syndrome (ASyS), which is a form of IIM that also develop ILD commonly but associated with a low risk of cancer (8, 13).

The risk of malignancy is highest within the first year following a DM diagnosis, with most cancers detected within three years; thereafter, the risk gradually declines (14). Consequently, current guidelines recommend extended cancer surveillance over 3 to 5 years for anti-TIF1γ-positive DM patients, using either conventional imaging or whole-body PET/CT (15, 16). The coexistence of cancer necessitates cautious use of immunosuppressive therapy for DM, requiring physicians to carefully prioritize which condition, malignancy or DM, should be managed first. Moreover, for patients with anti-TIF1γ-positive DM, the presence of cancer is the most significant determinant of survival (17, 18). Thus, decoding precise immune failures enabling tumor immune escape is meaningful for those patients with anti-TIF1γ-positive DM.

While muscle biopsy remains the diagnostic gold standard for DM, peripheral blood has emerged as a critical, less invasive source for identifying biomarkers and elucidating pathophysiology (e.g., MSA tests). Human peripheral blood mononuclear cells (PBMCs) serve as a sentinel population that responds to various pathophysiological stimuli through specific immunological changes and shifts in immunophenotype (19). Single-cell RNA sequencing (scRNA-seq) offers a powerful, unbiased approach to dissecting these immune responses, which enable the simultaneous characterization of alterations in both cellular composition and molecular signatures. In the context of anti-TIF1γ-positive DM, applying scRNA-seq to PBMCs may provide critical insights into how the immune system permits or promotes cancer progression.

However, the systemic immune landscape of anti-TIF1γ-positive DM patients remains poorly defined. To address this gap and identify potential drivers of cancer risk, we generated a single-cell atlas of PBMCs from anti-TIF1γ-positive DM patients and healthy controls. To further delineate subtype-specific immune signatures, we incorporated PBMC data from anti-MDA5-positive DM and ASyS patients as disease controls with low cancer risk (12, 13). Our analysis revealed key immune alterations in anti-TIF1γ-positive DM, including: (1) reduced frequencies of cytotoxic populations (CD8^+^ T cells and NK cells) accompanied by increased of B and plasma cell compartments; (2) reduced phagocytic capacity in non-classical CD16^+^ monocytes. Most notably, we observed a selective increase in Treg frequency in anti-TIF1γ-positive DM that was not present in other IIM subtypes. Together, this high-resolution atlas maps immune dysregulation in anti-TIF1γ-positive DM and provides a foundation for future investigations into the relationship between these immune perturbations and malignancy risk.

## Results

### Cell typing of PBMCs in anti-TIF1γ-positive DM relative to HC through scRNA-seq

To delineate peripheral immune characteristics of anti-TIF1γ-positive DM patients, we performed scRNA-seq against PBMCs from five DM (DM1-DM5), and five healthy subjects (HC1-HC5) (**Figure 1a**). Clinical characteristics of all participants are detailed in **Supplementary Table S1**. Data from three cases of anti-MDA5-positive DM and three cases of ASyS patients were also incorporated (**Figure 1a**) (12, 13). Our dataset comprised 796,368,124 transcripts across 185,619 cells, with a median detection of 1,745 genes per cell (**Supplementary Figure S1**). Principal component analysis of the top 3,000 differentially expressed genes (DEGs) identified 28 distinct cell clusters, which were further annotated against established immune lineages using canonical marker genes (**Supplementary Figures S2a–c**). Following neutrophil filtering and doublet removal, 165,354 high-quality cells were retained yielding representing all six major PBMC subtypes (**Figures 1b, c**; **Supplementary Figure 2d**), with a ranked breakdown into 51,290 myeloid cells (31.02%), 41,472 CD4^+^ T cells (25.08%), 31,944 CD8^+^ T cells (19.32%), 26,860 NK cells (16.24%), 12,677 B cells (7.67%), and 1,111 plasma cells (0.67%).

**Figure 1:**
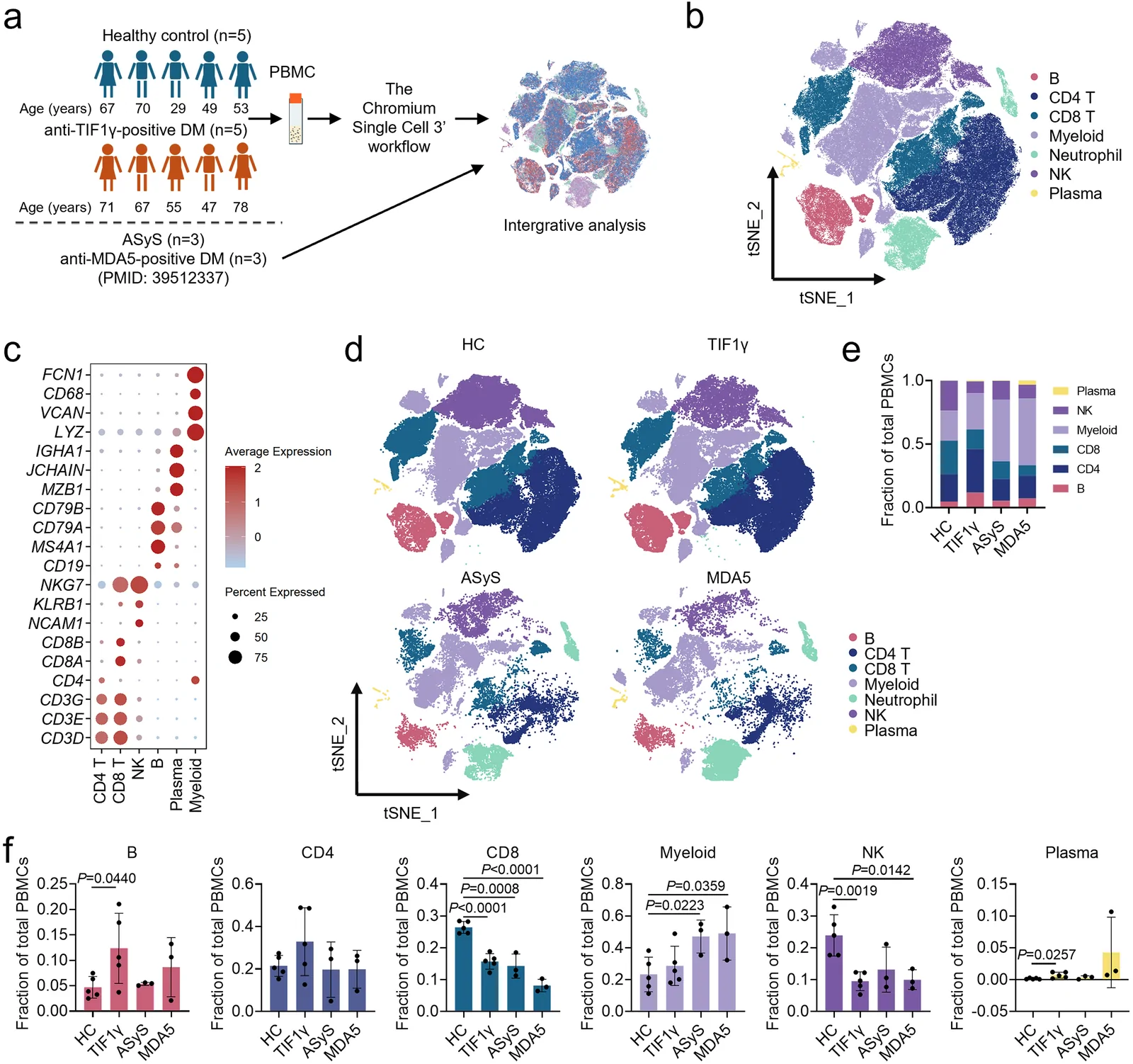
Cell typing of PBMCs in anti-TIF1γ-positive DM relative to HC group through scRNA-seq. **(a)** Flowchart depicting the overall experimental design of this study. **(b)** tSNE plots of the 165,354 cells (exclude neutrophil) profiled with each cell color-coded for cell types. **(c)** Dot plots of cell type marker genes. Expression values were normalized and scaled to the averages. Neutrophils are not shown here. **(d)** tSNE plots of the seven major cell types split by groups (HC, TIF1γ, ASyS and MDA5), color-coded by cell types. Neutrophils are also included. **(e)** Stacking plots of major cell types in the four groups. **(f)** Proportions of each PBMC cell subtypes in each sample, grouped by HC TIF1γ, ASyS and MDA5 samples. Data are mean ± SD, student *t* test.

Analysis of overall PBMC composition revealed homogeneous populations among HC but distinct heterogeneity within the anti-TIF1γ-positive DM group (**Figures 1d, e**; **Supplementary Figure 2e**). Compared to HCs, the anti-TIF1γ-positive DM group exhibited significant shifts in specific immune subsets: proportions of cytotoxic cells were significantly decreased, while B-lineage cells were increased (**Figure 1f**). Specifically, CD8^+^ T cells (HC: 26.33% vs. anti-TIF1γ-positive DM: 15.55%; *p* < 0.0001) and NK cells (HC: 23.56% vs. anti-TIF1γ-positive DM: 9.34%; *p* = 0.0019) were decreased in anti-TIF1γ-positive DM. In contrast, B cells (HC: 4.86% vs. anti-TIF1γ-positive DM: 11.93%; *p* = 0.0440) and plasma cells (HC: 0.15% vs. anti-TIF1γ-positive DM: 0.72%; *p* = 0.0257) were higher (**Figure 1f**). The decreasing of CD8^+^ T cell proportions in total PBMC is a common character in these three IIM subtypes (**Figure 1f**). Although an increase in B cell lineage was observed in anti-MDA5-positive DM and ASyS cases, the increase was not statistically significant (**Figure 1f**). The specific expansion of circulating myeloid cells, primarily monocytes in PBMCs, was consistent with previous reports in anti-MDA5-positive DM (11, 20). No differences were observed between anti-TIF1γ-positive DM and healthy controls of myeloid cells in PBMCs (**Figure 1f**).

We next examined TRIM33 (the gene encoding TIF1-γ) expression across six major immune cell types. Notably, TRIM33 expression was significantly decreased specifically in plasma cells from anti-TIF1-γ-positive DM patients, whereas no significant differences were observed in B cells, CD4^+^ T cells, CD8^+^ T cells, myeloid cells, or NK cells compared with healthy controls (**Supplementary Figure S3**).

Collectively, these findings demonstrate distinct peripheral immune remodeling patterns in anti-TIF1γ-positive DM, generally characterized by attenuated cytotoxic responses and enhanced humoral immunity.

### Flow cytometry validates altered frequencies of PBMC subtypes in anti-TIF1γ-positive DM patients compared to healthy controls

To verify the alterations in lymphocyte subtypes identified by scRNA-seq (**Figure 1f**), an expanded cohort (HC, n = 40; anti-TIF1γ-positive DM, n = 21) (**Supplementary Table S2**) was collected, and the proportions of CD4^+^ T, CD8^+^ T, B, and NK cells were confirmed by flow cytometry (**Figures 2a–d**). The protein-level data further confirmed a decrease in cytotoxic cells (CD8^+^ T and NK cells) and an increase in B cells within the total PBMC population (**Figures 2a–d**). Of note, a statistically significant elevation of the CD4/CD8 ratio was observed in anti-TIF1γ-positive DM patients compared with healthy controls, indicating dysregulation of CD4/CD8 homeostasis in this disease subtype (**Figure 2e**).

**Figure 2:**
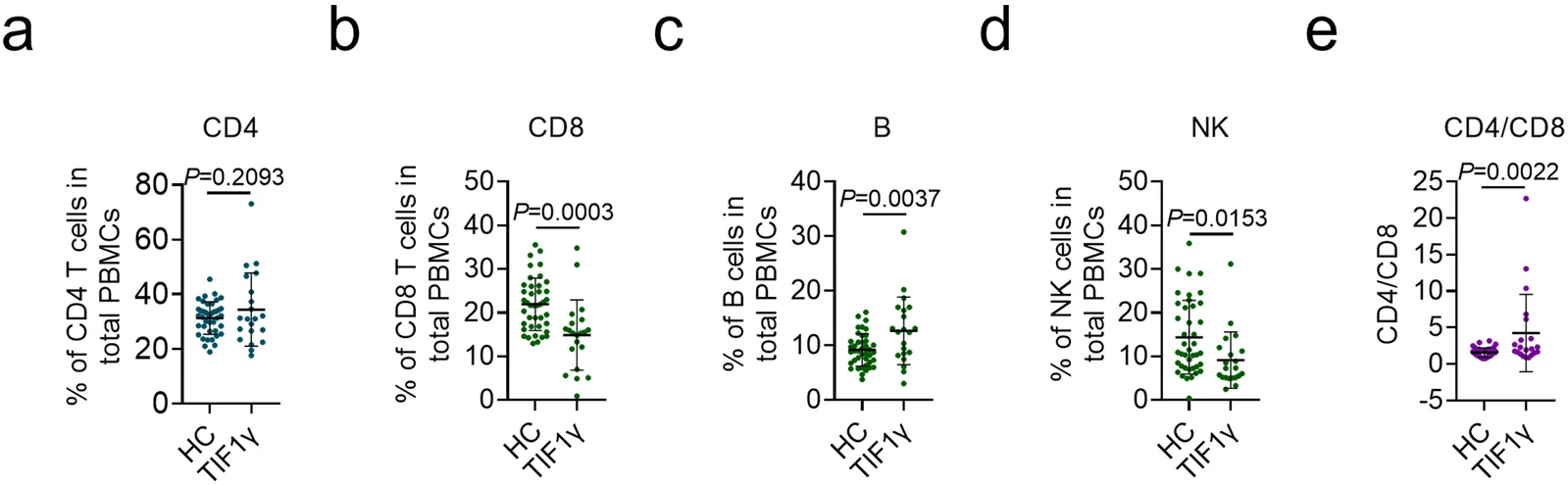
Flow cytometry validates altered frequencies of PBMC subtypes in anti-TIF1γ-positive DM patients compared to healthy controls. **(a–d)** Proportions of CD4^+^ T cells (CD3^+^CD4^+^, **(a)**, CD8^+^ T cells (CD3^+^CD4^+^, **(b)**, B cells (CD45^+^CD19^+^, **(c)** and NK cells (CD3^-^CD56^+^, **(d)** in total PBMCs, determined by flow cytometry. HC, n=40; TIF1γ DM, n=21. Data are mean ± SD, student *t* test. **(e)** The ratio of CD4^+^ T cells and CD8^+^ T cells were calculated according the data from **(a, b)**. Data are mean ± SD, student *t* test.

### Reduced cytotoxic cell frequency with enhanced IFN signature in anti-TIF1γ-positive DM

The reduction of circulating CD8^+^ T and NK cells is common across all three IIM subtypes (**Figures 1f**, **2b, d**). This consistent finding suggests that these changes may represent a shared feature of IIM rather than a TIF1γ-specific mechanism. Nevertheless, CD8^+^ T cells have been shown to be critical for the development of experimental myositis in mouse models induced by immunogenic TIF1γ (21). We therefore investigated whether the alteration of CD8^+^ T cells observed in our data contributes to the pathogenesis of anti-TIF1γ-positive DM.

We further performed unsupervised clustering of CD8^+^ T cells, identifying 9 distinct clusters (**Supplementary Figure S4a**). These subtypes were annotated using canonical marker genes into four functionally defined subsets (**Figures 3a, b**; **Supplementary Figures 4a, b**) (22). All four groups contributed to each CD8^+^ subtype (**Figure 3c**). Notably, despite the overall decrease in CD8^+^ T cell proportions across all three IIM subtypes (**Figures 1f**, **2b**), not all four CD8^+^ T cell subsets were statistically decreased. Indeed, it was mainly the reduced frequency of the CD8^+^ effector memory T (TEM) cell subtype that contributed to the statistically significant decrease of CD8^+^ T cell proportions in anti-TIF1γ-positive DM (**Figure 3d**). This was not the case in anti-MDA5-positive DM or ASyS, in which CD8^+^ naïve, CD8^+^ central memory T (TCM), and CD8^+^ TEM subtypes were commonly decreased, along with an increase in proliferating CD8^+^ T cells (**Figure 3d**). Thus, the deficit of CD8^+^ TEM cells is the distinguishing feature of circulating CD8^+^ T cell reduction in anti-TIF1γ-positive DM.

**Figure 3:**
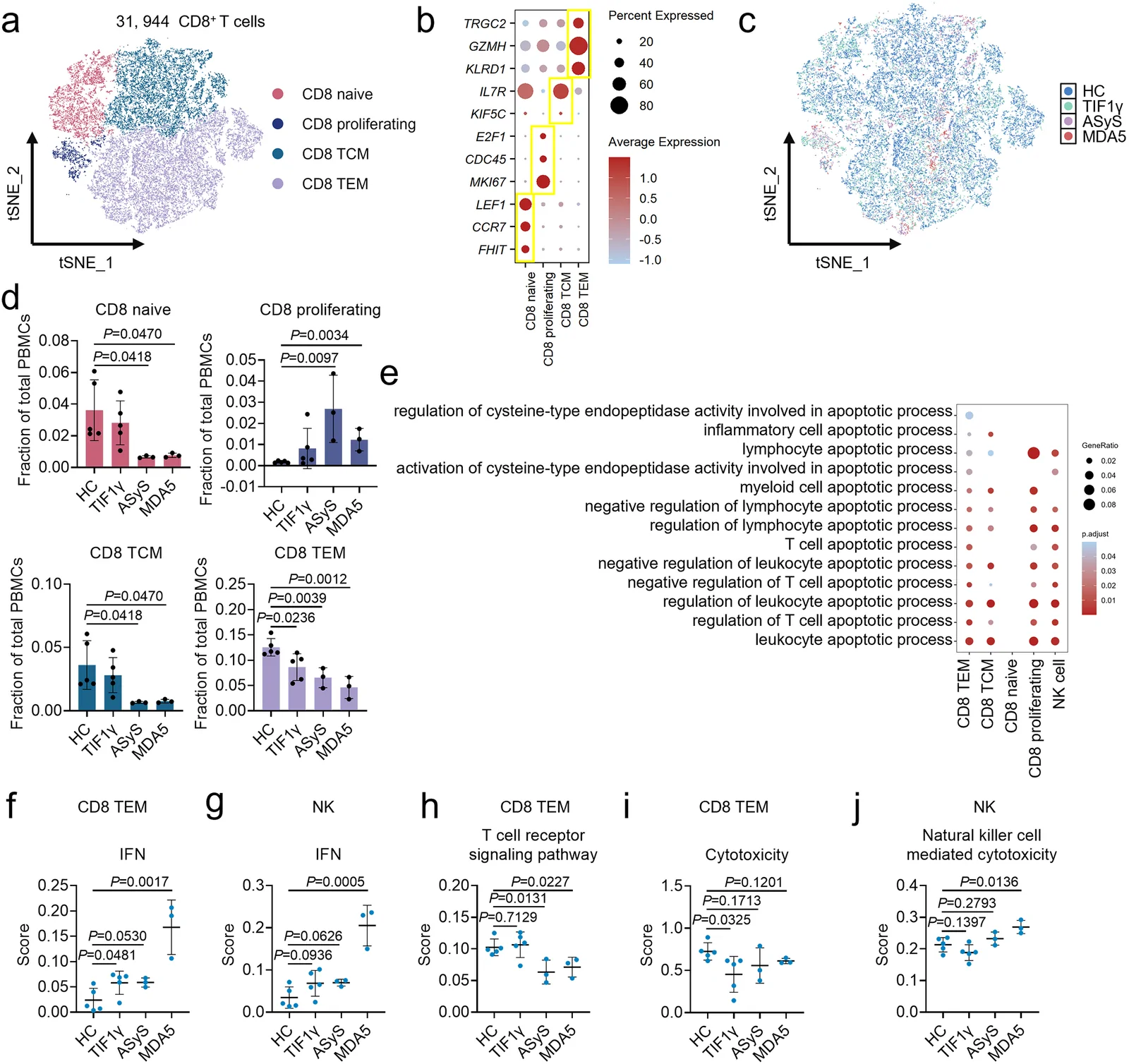
Reduced cytotoxic cell frequency with enhanced IFN signature in anti-TIF1γ-positive DM. **(a)** tSNE plots of the four CD8^+^ T cell subtypes with each cell color-coded for the associated subtypes. **(b)** Dot plots of CD8^+^ T cell subtype marker genes. Expression values were normalized and scaled to the averages. **(c)** tSNE plot of the CD8^+^ T cell, color-coded by cell groups (HC, TIF1γ, ASyS and MDA5). **(d)** Proportions of each CD8^+^ T cell subtypes in each sample, grouped by HC, TIF1γ, ASyS and MDA5 samples. **e**) Representative Gene Ontology (GO) pathways associated with cell death that enriched by the highly expressed genes in anti-TIF1γ-positive TEM subsets. Dot size indicates gene ratio, and color represents adjusted *p* values. **(f, g)** IFN scores in CD8^+^ TEM **(f)** and NK **(g)** subsets in the four groups (HC, TIF1γ, ASyS and MDA5). **(h)** T cell receptor signaling pathway scores in CD8^+^ TEM subsets in the four groups (HC, TIF1γ, ASyS and MDA5). **(i)** Cytotoxicity scores in CD8^+^ TEM subsets in the four groups (HC, TIF1γ, ASyS and MDA5). **(j)** Nature Killer cell mediated cytotoxicity pathway scores in NK subset in the four groups (HC, TIF1γ, ASyS and MDA5). Data **(d, f–i)** are mean ± SD, student *t* test.

To explore potential mechanisms underlying the reduced frequency of CD8^+^ TEM cells in anti-TIF1γ-positive DM (**Figure 3d**), we analyzed DEGs in these populations compared to healthy controls (**Supplementary Figure S4c**). Enrichment analysis of upregulated genes in anti-TIF1γ-positive DM revealed an association with 13 cell death-related pathways (**Figure 3e**). Notably, most of these apoptosis signals were also enriched in NK cells, as well as in CD8^+^ TCM and proliferating CD8^+^ T cells, but none of them were enriched in naïve cells (**Figure 3e**). To exclude the possibility that the observed apoptosis-related signatures were driven by differential cell quality or stress, we compared key QC metrics, including nCount_RNA, nFeature_RNA and mitochondrial percentage, between HC and TIF1γ DM within each subset. No consistent decreasing of transcripts and increasing of mitochondrial percentage were detected (**Supplementary Figures S4d–f**), suggesting that the transcriptional enrichment of apoptotic pathways is unlikely to reflect technical artifacts. We next compared apoptosis pathway scores across CD8^+^ subsets to assess whether this enrichment was specific to TEM cells. Indeed, the enrichment was most pronounced in CD8^+^ TEM cells with 11 out of these 13 pathways showed significantly higher pathway scores than both CD8^+^ TCM and proliferating CD8^+^ T cells (**Supplementary Figure S4g**). This subset-specific enrichment may help explain their selective reduction. Nevertheless, these data remain correlative; functional validation of increased apoptosis is required to establish a causal link between this transcriptional signature and the reduced frequencies of these populations.

Having confirmed the reduction characteristics of cytotoxic cells in anti-TIF1γ-positive DM, we next sought to define its immune molecular basis, focusing on CD8 TEM and NK cells. The increase in IFN score was most prominent and statistically robust in CD8^+^ TEM cells, whereas a similar, albeit statistically non-significant, trend was observed in NK cells (**Figures 3f, g**). The T cell receptor (TCR) signaling pathway initiates a cascade of molecular events that drive T cell activation and subsequent immune responses (23). To assess functional differences between disease states, we quantified TCR signaling pathway activity (KEGG: hsa04660) in CD8 TEM populations (**Figure 3h**). Anti-MDA5-positive DM and ASyS patients showed significantly lower pathway activity scores compared to HC, indicating reduced CD8^+^ T cell activation. However, this was not the case in anti-TIF1γ-positive DM (**Figure 3h**). In addition to TCR signaling, we assessed cytotoxic function in CD8^+^ TEM cells using a curated cytotoxicity gene expression score encompassing granzyme/perforin-mediated killing and effector molecules. (24, 25). Notably, CD8^+^ TEM cells from anti-TIF1γ DM patients exhibited significantly reduced cytotoxicity scores compared to HC (**Figure 3i**). Meanwhile, the cytotoxic capacity of NK cells remained unaltered, as evidenced by comparable NK cell-mediated cytotoxicity pathway scores (hsa04650) (**Figure 3j**).

Taken together, our data point to significantly reduced cytotoxic cell frequencies in anti-TIF1γ-positive DM, characterized by a loss of CD8^+^ TEM and NK cells (**Figures 1f**, **2b, d**), which may be associated with apoptosis-related transcriptional signatures (**Figures 3e**; **Supplementary Figure S4g**). However, the shared nature of cytotoxic cell reduction across IIM subtypes cautions against attributing anti-TIF1γ-related cancer risk solely to this feature.

### Increased naïve and intermediate B cell frequency in peripheral blood of anti-TIF1γ-positive DM patients

Compared to HCs, the number of circulating B cells and plasma cells showed an increasing trend in the anti-TIF1γ-positive DM group (*p* = 0.0440 for B cells; *p* = 0.0257 for plasma cells; **Figure 1f**). Flow cytometric analysis also confirmed a statistically significant increase in total B cells (CD45^+^CD19^+^) in the anti-TIF1γ-positive DM group (**Figure 2c**). To refine our B-cell analysis, we performed comprehensive annotation of B-cell subsets using established marker genes guided by literature (22, 26–29). tSNE plots further delineated B cell subsets, identifying intermediate, naïve, memory B cells and plasmablast (**Supplementary Figures S5a, b**, **4a, b**). We found that the increase in total B cells in the anti-TIF1γ-positive DM group was primarily associated with naive and intermediate B cells, which were elevated in both absolute cell counts and proportions (**Figures 4c–g**; **Supplementary Figure 5c**).

**Figure 4:**
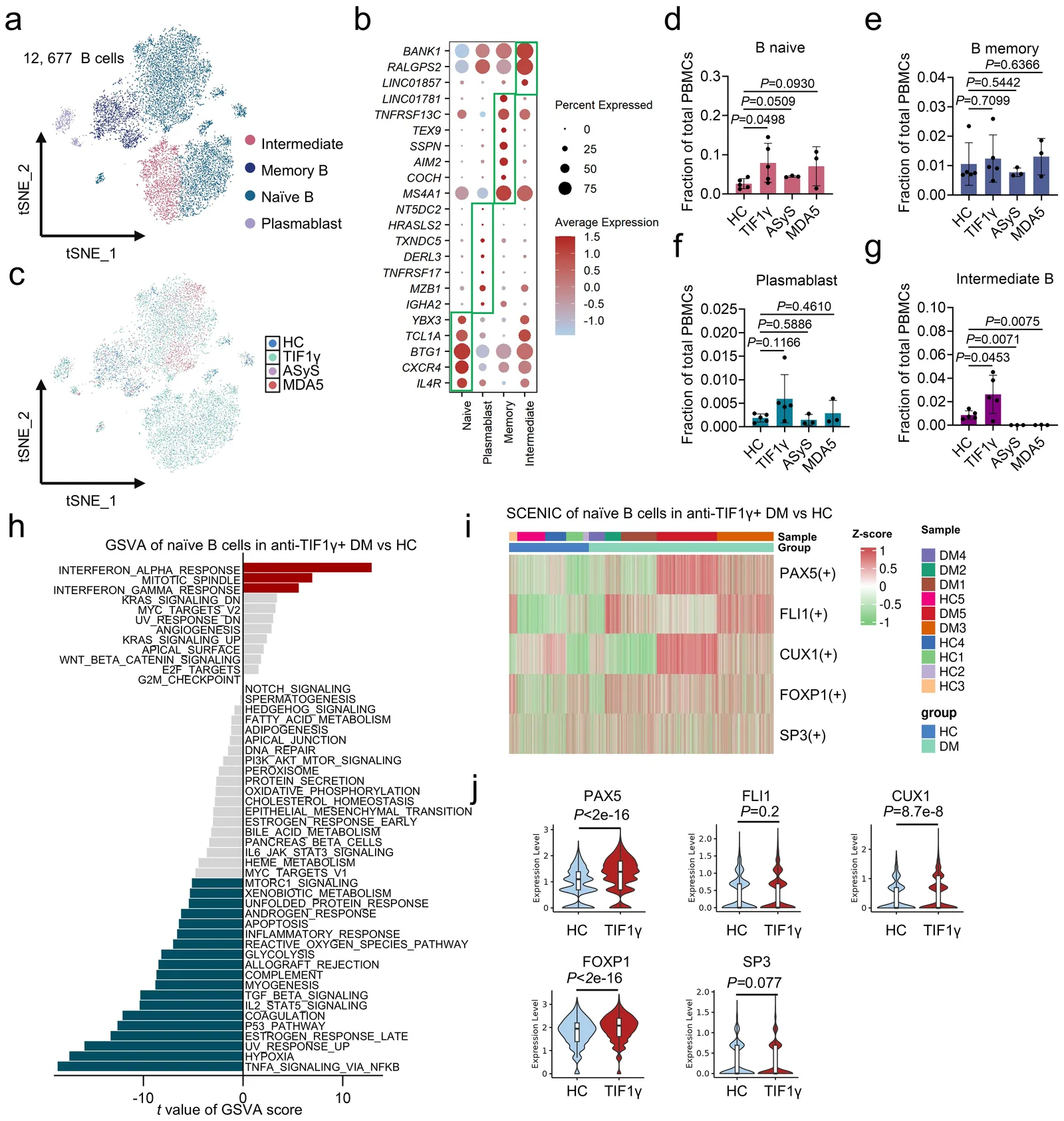
Increased naïve and intermediate B cell frequency in peripheral blood of anti-TIF1γ-positive DM patients. **(a)** tSNE plot of B cell subtypes with each cell color-coded for the associated subtypes. **(b)** Dot plots of B-lineage cell subtype marker genes. Expression values were normalized and scaled to the averages. **(c)** tSNE plot of the B cell, color-coded by cell groups (HC, TIF1γ, ASyS and MDA5). **(d–g)** Proportions of each B cell subtypes in each sample, grouped by HC, TIF1γ, ASyS and MDA5 samples. **(h)** Differential expression of hallmark pathway gene signatures scored per cell using gene set variation analysis (GSVA) of naïve B cells in anti-TIF1γ-positive DM versus HC. The data shown are *t* values from a linear model Box plots of UMIs detected in naïve B cells. **(i)** Heatmap displaying the area under the curve (AUC) scores for transcription factor binding motifs in anti-TIF1γ-positive DM naïve B cells compared to HC naïve B cells, as determined by SCENIC. The data highlights the top five transcription factors with the highest z-scores in DM naïve B cells. **(j)** Violin plots of the expression distribution of PAX5, FLI1, CUX1, FOXP1 and SP3 in the two groups (HC and anti-TIF1γ-positive DM) of naïve B cells. Data **(d–g, j)** are mean ± SD, student *t* test or Wilcoxon rank-sum test.

To investigate the basis for increased naïve and intermediate B cell frequency, we performed gene set variation analysis (GSVA) using hallmark pathway gene signatures. This revealed significant enrichment in the anti-TIF1γ-positive DM group for proliferative pathways, which including mitotic spindle (**Figures 4h**; **Supplementary Figure 5d**). Additionally, interferon pathways were more active in anti-TIF1γ-positive DM naive and intermediate B cells (**Figures 4h**; **Supplementary Figure 5d**).

To elucidate the mechanisms driving the widespread transcriptional and proliferative changes in anti-TIF1γ-positive DM, we performed single-cell regulatory network inference and clustering (SCENIC) analysis on naïve B cells to identify active transcription factors (TFs) and their regulatory networks (30). This analysis revealed 12 upregulated regulons in DM and the top five, namely PAX5(+), FLI1(+), CUX1(+), FOXP1(+) and SP3(+) (**Figure 4i**). PAX5, FLI1, and FOXP1 are established critical regulators of B cell development and function (31–34). Indeed, both PAX5 and FOXP1 were upregulated in naïve B cells in anti-TIF1γ-positive DM than in HC (**Figure 4j**). Moreover, these two transcripts were also consistently strong enriched across most of the naïve B cells in anti-TIF1γ-positive DM (**Figure 4j**). Together, these findings imply that these TFs, particularly PAX5 and FOXP1, may act in concert to drive the substantial increase in transcriptional activity within naïve B cells in anti-TIF1γ-positive DM (**Figures 4i, j**). Of course, further experimental validation to be performed to verify this hypothesis.

### Non-classical monocytes in anti-TIF1γ-positive PBMCs show a reduced proportion and impaired phagocytic capacity

Cells of the myeloid lineage (51,290 monocytes and DCs) represented the largest cell population in our dataset, although their proportions did not differ between the anti-TIF1γ-positive DM and HC groups (**Figure 1f**). However, the elevations in myeloid lineage in anti-MDA5-positive DM and ASyS were negligible (**Figure 1f**). Unsupervised PCA analysis classified these cells into 11 clusters (**Supplementary Figure S6a**), which were further rationalized into five subtypes (35): classical CD14 (CD14^+^CD16^−^) monocytes (clusters 1, 2, 3, 6, 7, 9 and 10), non-classical CD16 (CD14^dim^CD16^+^) monocytes (cluster 5), intermediate (CD14^+^CD16^+^) monocytes (cluster 4), conventional dendritic cells (cDCs, cluster 8), and plasmacytoid DCs (pDCs, clusters 11; **Figure 5a**, b and S6b). Interestingly, unlike anti-MDA5-positive DM cases, which show increased proportions of CD14^+^ monocytes (10), we did not detect differences in CD14^+^ monocytes between the anti-TIF1γ-positive DM and HC groups (**Figures 5c, d**). We caution against overinterpreting this finding as an explanation for the lower ILD risk in anti-TIF1γ DM. ILD pathogenesis is complex and differs substantially across anti-MDA5 DM, ASyS, and other IIM subtypes, involving type I IFN responses, macrophage polarization, and T-cell infiltration in addition to monocyte dynamics (12, 13). Indeed, recent evidence has strongly implicated inflammatory classical monocyte-like populations in DM pathogenesis (36–38).

**Figure 5:**
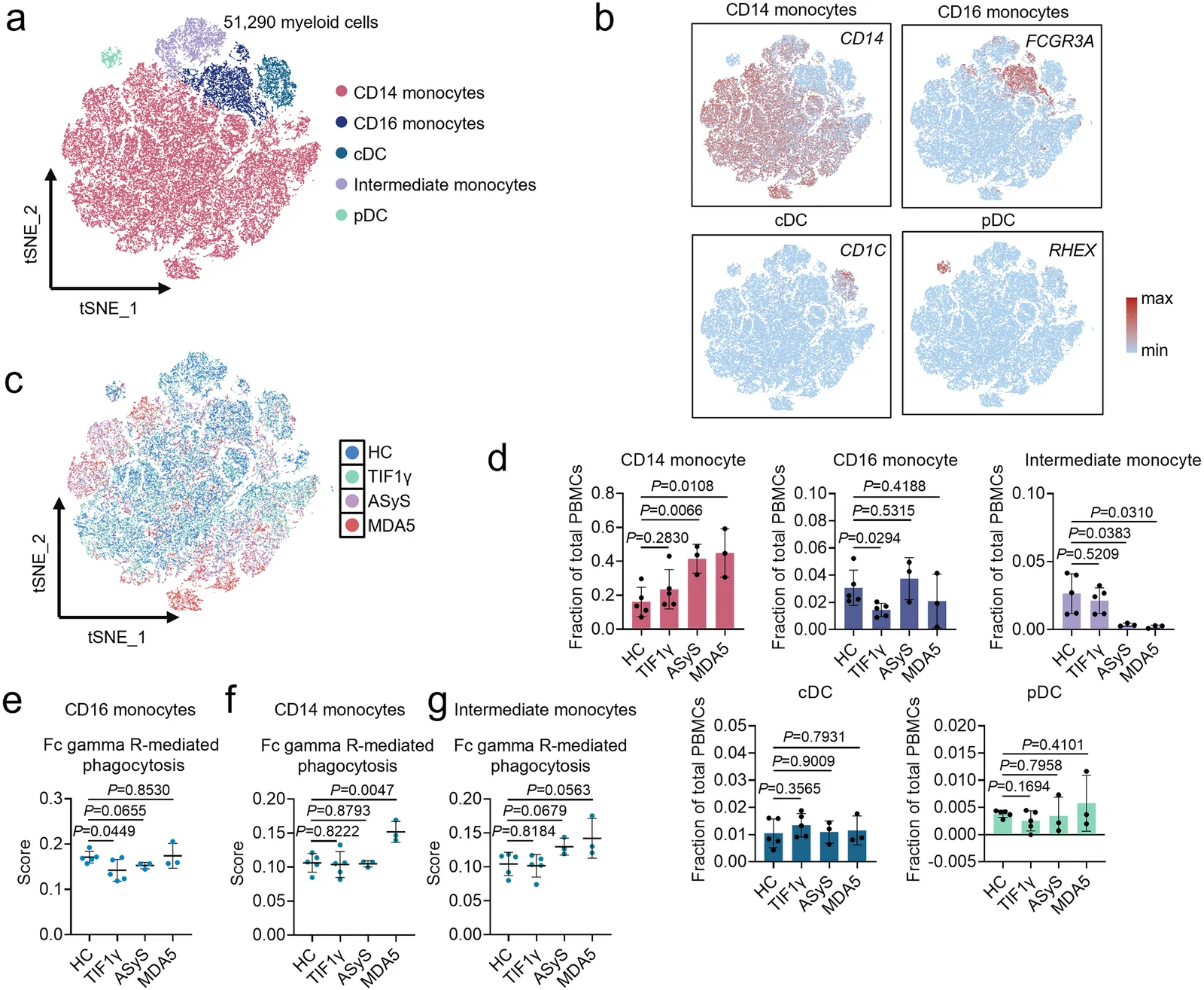
Non-classical monocytes in anti-TIF1γ-positive PBMCs show a reduced proportion and impaired phagocytic capacity. **(a)** tSNE plot of DC and monocytes subtypes with each cell color-coded for the associated subtypes. **(b)** tSNE plot of monocytes and DCs with each cell color-coded for the expression of a marker gene for the individual cell subtypes. **(c)** tSNE plot of DC and monocytes subtypes, color-coded by the four groups (HC, TIF1γ, ASyS and MDA5). **(d)** Proportions of each DC and monocytes subtypes in each sample, grouped by the four groups (HC, TIF1γ, ASyS and MDA5). **(e–g)** Fc gamma R-mediated phagocytosis scores in CD16 **(e)**, CD14 **(f)** and intermediate **(g)** monocytes in the four groups (HC, TIF1γ, ASyS and MDA5). Data **(d, e)** are mean ± SD, student *t* test.

In contrast, a decrease in CD16^+^ monocytes was evident, with proportions specifically reduced only in the anti-TIF1γ-positive DM group (**Figure 5d**). Human CD16^+^ monocytes are known to promote leukocyte recruitment to inflammatory sites and mediate potent antibody-dependent cellular cytotoxicity (ADCC) against cancer cells (39). Whether the specific alteration we observed here represents a risk factor for tumor development remains unknown. Consistent with a potentially compromised function, the decreased proportion of CD16^+^ monocytes in our anti-TIF1γ-positive DM cohort were accompanied by reduced scores for Fc gamma receptor-mediated phagocytosis (**Figure 5e**). While classical and intermediate monocytes displaying no significant difference (**Figures 5f, g**). These results suggest that the downregulation of this pathway may be relatively enriched in nonclassical monocytes, although functional phagocytosis assays are required to confirm this transcriptional finding (**Figure 5e**).

### Increased frequency of regulatory T cells in anti-TIF1γ-positive DM

CD4^+^ T cells (41,472 cells) constituted the second largest cell subpopulation in our dataset. Dimensionality reduction of CD4^+^ T cells revealed 17 clusters (**Supplementary Figure S7a**), which were further classified into four subtypes based on classical markers (**Supplementary Figures S7b**, **6a, b**): TEM, TCM, naïve, and regulatory T cells (Tregs) (**Figures 6a, b**). While the proportions of CD4^+^ naïve and memory T cells in anti-TIF1γ-positive DM patients were comparable to those in HCs, Tregs were specifically increased in anti-TIF1γ-positive DM patients (**Figures 6c–f**; **Supplementary Figure 7c**).

**Figure 6:**
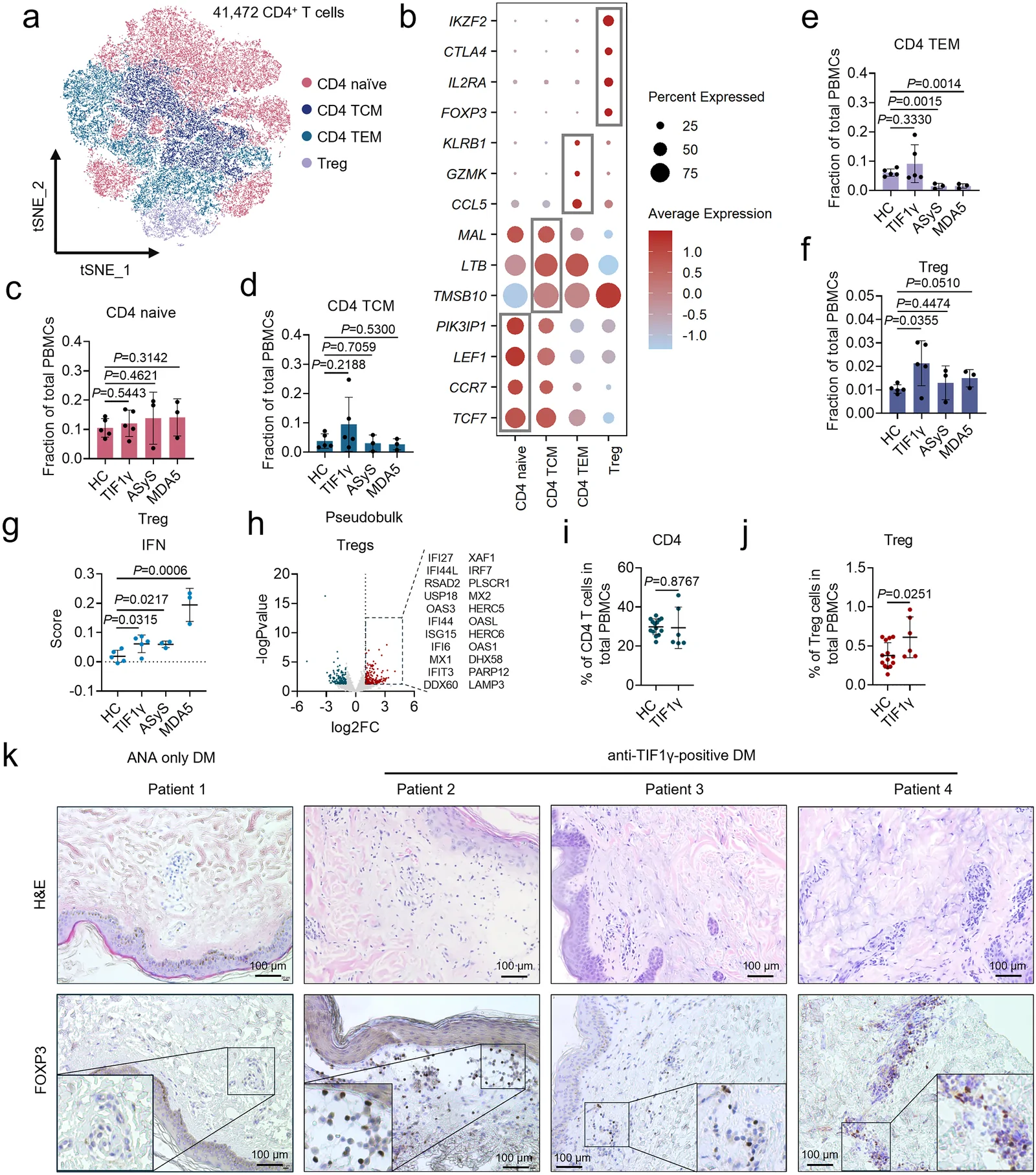
Increased frequency of regulatory T cells in anti-TIF1γ-positive DM. **(a)** tSNE plot of CD4^+^ T cells subtypes with each cell color-coded for the associated subtypes. **(b)** Dot plots of CD4^+^ T cells subtypes marker genes. Expression values were normalized and scaled to the averages. **(c–f)** Proportions of each CD4^+^ T cells subtypes in each sample, grouped by the four groups (HC, TIF1γ, ASyS and MDA5). **(g)** IFN scores in Treg in the four groups (HC, TIF1γ, ASyS and MDA5). **(h)** Volcano plot of the differential expression genes of Treg cells in anti-TIF1γ-positive DM versus HC. IFN-inducible genes are also indicated. The DEGs were calculated by pseudotime analysis. **(i, j)** Proportions of CD4^+^ T cells and Treg cells in total PBMCs, determined by flow cytometry. **(k)** Representative microphotographs of IHC staining of FOXP3 in skin tissue sections of an antinuclear antibody (ANA) only DM and three anti-TIF1γ-positive DM. Routine hematoxylin and eosin (H&E) staining were also shown. Scale bar, 100 µm. Data **(c-f, g, i, j)** are mean ± SD, student *t* test.

To investigate potential upstream mechanisms, we performed SCENIC analysis. This identified several upregulated regulons in anti-TIF1γ-positive DM Tregs, including IRF7(+), BDP1(+), STAT1(+), USF1(+), ZBTB40(+), and IKZF2(+) (**Supplementary Figure S7d**). However, none of these transcription factor genes were themselves highly expressed or upregulated in anti-TIF1γ-positive DM Tregs at the transcript level (**Supplementary Figure S7e**). Thus, we did not identify a clear transcriptional driver accounting for the increased Treg proportion in anti-TIF1γ-positive DM.

We therefore employed CellChat to explore cell-cell communication among the elevated naïve B cells, plasma cells, and Tregs (40). This approach provides hypothesis-generating ligand-receptor predictions. We focused on the ADGRE5, SELPLG, and ITGB2 signals, as these three pathways exhibited differential outgoing or incoming signals from or to Tregs between anti-TIF1γ-positive DM and HC groups (**Supplementary Figure S8a**). Of note, a specific ITGB2 signal from naïve B cells to Tregs was detected in anti-TIF1γ-positive DM patients but not in healthy controls (**Supplementary Figure S8a**). Indeed, ITGB2 has been reported to play a critical role in regulating Treg development, immune homeostasis, and suppressive function (41–43). To our knowledge, no prior direct evidence supports this specific interaction in the context of IIM or anti-TIF1γ DM. We therefore present this observation as a candidate pathway for future investigation, not as a mechanistic conclusion.

We further evaluated Treg suppressive potential using a gene-expression scoring approach. Based on prior reports that Treg subsets with high expression of MHC class II molecules (CIITA, HLA-DRA, HLA-DRB1, HLA-DRB3, HLA-DRB4, HLA-DRB5, HLA-DQA1, HLA-DQA2, HLA-DQB1, HLA-DQB2, HLA-DQB3, HLA-DPA1, HLA-DPB1) or co-expression of CD39 and CCR7 (ENTPD1, CCR7) exhibit enhanced suppressive activity (44–46), we compiled a panel of these genes and scored Treg suppressive potential (**Supplementary Figure S8b**). Using this approach, we observed a slight increase in Treg suppressive capacity in anti-TIF1γ DM patients. However, we acknowledge that gene-expression scoring cannot substitute for functional validation, and the *in vitro* suppression assay could further support this conclusion.

Tregs suppress immune function through both cell-contact mechanisms and the secretion of inhibitory cytokines (47). Previous studies have demonstrated that Treg depletion in mice triggers widespread autoimmune diseases (48, 49). We propose a working hypothesis that distinguishes two temporal layers of Treg function in this paraneoplastic context. In the early phase, Treg expansion may primarily represent a reactive compensatory mechanism triggered by tumor-associated antigen exposure, aimed at limiting collateral tissue damage. However, in the setting of chronic tumor persistence, we speculate that sustained Treg accumulation may progressively adopt a permissive role in tumor progression. This putative transition remains conceptual and requires experimental validation (50).

Given that an active interferon (IFN) signature is a hallmark of anti-TIF1γ DM, we explored this signature specifically within the Treg compartment to provide additional context for this seemingly unexpected phenomenon. Similar to other PBMC subtypes in DM, all CD4^+^ T cell subtypes exhibited elevated type I IFN activity (**Figure 6g**; **Supplementary Figure 8c**). In support of this, multiple IFN-inducible genes were found to be elevated in anti-TIF1γ-positive DM Tregs compared with their healthy control counterparts (**Figure 6h**; **Supplementary Figure 8d**). Furthermore, akin to naïve B cells (**Figure 4h**), Tregs from anti-TIF1γ-positive DM patients showed upregulation of pathways involved in cell proliferation, including the mitotic spindle and KRAS signaling pathways (**Supplementary Figure S8c**).

Considering the critical role of Tregs in tumor progression (51), we further confirmed an increased proportion of Tregs within total PBMCs in anti-TIF1γ-positive DM patients using flow cytometry (**Figures 6i, j**; **Supplementary Table S3**). Human peripheral blood acts as the body’s central highway, connecting all organs, and PBMCs within it both respond to soluble inflammatory mediators released from any tissue and exchange immune cells with inflamed organs. We therefore examined Tregs residing in the skin tissue of anti-TIF1γ-positive DM patients. Abundant Tregs were detected in multiple regions, including within infiltrating immune cell populations and in the spaces between the epidermis and dermis (**Figure 6k**). In contrast, few Foxp3^+^ cells were observed in a low-risk malignancy case of connective tissue disease where only anti-nuclear antibody was positive (**Figure 6k**).

## Discussion

Our study establishes the first single-cell transcriptomic atlas of PBMCs from anti-TIF1γ-positive DM patients, revealing distinct immune states. Despite significant depletion of CD8^+^ T and NK cells, the comparable proportions of these cytotoxic cells among anti-TIF1γ-positive DM, anti-MDA5-positive DM, and ASyS suggest that this is a shared feature across IIM subtypes rather than a TIF1γ-specific mechanism. We also uncovered a reduced proportion and impaired phagocytic capacity of CD16^+^ monocytes. In contrast, circulating B-lineage cells, including naïve B cells, intermediate B cells and plasma cells, were markedly increased. Most importantly, an increased proportion of Tregs was specifically confirmed in anti-TIF1γ-positive DM. We hypothesize that this Treg elevation may contribute to an immunosuppressive milieu, but this remains speculative and requires functional validation in prospective cohorts. Together, these results propose a specific immunological landscape in anti-TIF1γ-positive DM, characterized by coexisting impaired cellular immunity, heightened humoral responses, and expansion of tumor-permissive Tregs.

While the peripheral immune profile of anti-MDA5-positive DM has been well characterized due to its strong association with rapidly progressive ILD (RP-ILD) and high mortality (10, 11, 20), the immunobiology of anti-TIF1γ-positive DM remains poorly understood. Intriguingly, these distinct clinical phenotypes align with different etiologic triggers: viral infections are implicated in anti-MDA5-positive DM pathogenesis (52), whereas anti-TIF1γ positivity shows a robust but mechanistically unexplained association with malignancy (6). Somatic DNA mutations have been identified as tumorigenic drivers in anti-TIF1γ-positive DM (7), consistent with observations that anti-TIF1γ-positive patients harbor more numerous tumor genetic alterations compared with seronegative cases (53). These mutations may generate altered proteins that serve as neoantigens, eliciting a sustained anti-tumor immune response (1). Through molecular mimicry, this response cross-reacts with endogenous proteins, manifesting an autoimmune response. The resulting immune pressure could lead to either: (1) effective tumor elimination via immunologic surveillance, or (2) immunoediting, whereby malignant cells adapt to evade detection, ultimately progressing to occult cancer (1, 54).

Our study now demonstrates that Treg cells, critical mediators of an immune-permissive environment conducive to tumor development, are present at increased frequency in anti-TIF1γ-positive DM alongside a hyperactive IFN signature. Indeed, a previous study reported that the number of Tregs in the peripheral blood of patients with DM was significantly reduced compared to healthy controls (55), a finding consistent with the established notion that Treg depletion in mice triggers widespread autoimmune diseases (48, 49). However, by increasing the resolution of DM subtype analysis and focusing specifically on anti-TIF1γ-positive DM, we arrived at a totally different conclusion: an increased proportion of Tregs in PBMCs was observed. This finding may appear counterintuitive to some readers, as Tregs are typically associated with immunosuppression.

We further evaluated Treg suppressive potential using a gene-expression scoring approach based on a panel of genes associated with enhanced suppressive activity (including MHC class II genes, CCR7, and ENTPD1) (44–46). Using this approach, we observed a slight increase in Treg suppressive capacity in anti-TIF1γ DM patients. However, we acknowledge that gene-expression scoring cannot substitute for functional validation, and the *in vitro* suppression assay should be employed for further investigation.

We propose a working hypothesis that distinguishes two temporal layers of Treg function in this paraneoplastic context. In the early phase, elevated Treg frequency may primarily represent a reactive compensatory mechanism triggered by tumor-associated antigen exposure, aimed at limiting collateral tissue damage. However, in the setting of chronic tumor persistence, we speculate that sustained Treg accumulation may progressively adopt a permissive role in tumor progression. This putative transition remains conceptual and requires experimental validation. Importantly, we emphasize that our current cross-sectional data only support association, not causation. Functional longitudinal studies and *in vivo* lineage-tracing or depletion experiments are required to validate this temporal switch.

Our finding of increased Treg frequency in anti-TIF1γ-positive DM appears paradoxical given that TIF1γ deficiency causes Treg instability and loss of Foxp3 expression during inflammation (56). However, this discrepancy underscores a fundamental distinction: anti-TIF1γ autoantibodies do not equate to cell-intrinsic loss of TIF1γ function in Tregs. Indeed, we observed no reduction in TRIM33 expression in CD4 T cells from these patients, suggesting intact TIF1γ activity (**Supplementary Figure S3**). The Treg expansion we detected likely reflects a reactive compensatory response to systemic inflammation rather than impaired TIF1γ-mediated stability. Nevertheless, the mechanistic insight that TIF1γ restrains β-catenin-TCF7 signaling to preserve Treg identity raises the possibility that chronic inflammation in anti-TIF1γ-positive DM could eventually compromise TIF1γ-dependent Treg stability over time. Whether this contributes to Treg dysfunction in affected tissues warrants further investigation.

A major limitation of our study is the exclusive inclusion of anti-TIF1γ-positive DM patients without documented malignancy at the time of sampling. Consequently, our findings should be interpreted strictly as an immunophenotypic characterization of the cancer-free subset, rather than as a mechanism underlying cancer susceptibility. We cannot determine whether the observed immune alterations are cancer-specific, cancer-predictive, or simply epiphenomena of the autoimmune process itself. In light of recent evidence demonstrating that anti-CCAR1 antibodies are preferentially associated with cancer-negative anti-TIF1γ DM (57), the immunological landscape may differ substantially between patients with and without concurrent malignancy. Thus, our data should be viewed as hypothesis-generating and require validation in prospective cohorts that include cancer-positive patients.

Our analysis confirmed the elevation of classical monocytes in anti-MDA5-positive DM and ASyS, both of which are associated with ILD (10, 11, 13). In contrast, the proportion of classical monocytes in anti-TIF1γ-positive DM was comparable to that in HCs. We caution against overinterpreting this finding as an explanation for lower ILD risk, as ILD pathogenesis is multifactorial and recent evidence has strongly implicated inflammatory classical monocyte-like populations in DM pathogenesis (36–38).

Lymphopenia is a well-established feature of autoimmune diseases like systemic lupus erythematosus and rheumatoid arthritis (58), and is frequently observed in DM, particularly in anti-MDA5 positive patients (59, 60). Consistent with this, we found significantly lower absolute lymphocyte counts in the anti-TIF1γ-positive DM group compared to healthy controls (**Supplementary Tables S2**, **S3**). One potential explanation is the migration of lymphocytes to affected muscle and skin sites due to autoinflammation. Indeed, patients with TIF-1γ autoantibodies have more extensive skin involvement (61). As the largest organ in the human body, extensive skin inflammation could sequester a large number of lymphocytes, predominantly from the peripheral blood, thereby leading to lymphopenia. Nonetheless, given the overall lymphopenia in peripheral blood, the decreased proportions of cytotoxic cells and CD16^+^ monocytes likely reflect reduction of absolute cell numbers.

Importantly, we note that the reduction in cytotoxic cell frequencies was observed across multiple IIM subtypes, indicating that this is not a unique feature of TIF1γ DM. Therefore, this alteration alone cannot explain the particularly elevated cancer risk associated with TIF1γ autoantibodies. Rather than being the sole or primary cause, we propose that cytotoxic reduction may represent a baseline vulnerability shared among IIM patients. In the specific context of TIF1γ DM, this baseline defect may be compounded by other concurrent immune abnormalities (e.g., increased Treg frequency, B-cell dysregulation) to collectively create a milieu more permissive for tumor emergence.

As a type of autoimmune disease, dysregulation of immune cells is a fundamental characteristic. Notably, anti-TIF1γ-positive DM patients exhibit greater immune heterogeneity than healthy controls, indicating significant interindividual variability despite sharing the same disease subtype (**Figures 2a-d**, **6i, j**). This may explain the variable clinical responses to the same treatment regimen.

### Study limitations

A limitation of our study is its reliance on peripheral immune profiling to infer pathogenic mechanisms in anti-TIF1γ-positive DM. Integrating single-cell data from the affected muscle and skin microenvironments would likely provide deeper insights, particularly into patterns of muscle-infiltrating immune cells. However, obtaining an adequate number of affected tissue samples from both healthy controls and DM patients has proven challenging, as most patients refused to undergo muscle biopsy. Blood draws were far more acceptable. Despite this limitation, we successfully collected a limited number of skin tissue samples from anti-TIF1γ-positive DM patients, in which enrichment of Tregs was confirmed by IHC.

Another limitation of this study is the inability to validate plasma cells by flow cytometry, despite their biological importance as the putative producers of anti-TIF1γ autoantibodies. In our scRNA-seq data, plasma cells were extremely rare in both anti-TIF1γ DM patients (0.714% ± 0.455%) and healthy controls (0.144% ± 0.107%), and their absolute counts in the flow validation cohort were too low for reliable quantification. Additionally, the viability of plasma cells following PBMC cryopreservation and thawing remains uncertain, rendering post-thaw flow cytometric analysis unreliable (62). A well-recognized challenge given that plasma cells predominantly reside in bone marrow and lymphoid organs rather than in peripheral blood (63). Nevertheless, our scRNA-seq identification relied on canonical markers (IGHA1, JCHAIN, MZB1), and the finding is biologically coherent with the validated B-cell expansion and elevated serum autoantibody titers in these patients. Future studies employing bone marrow sampling or plasma cell enrichment strategies are warranted to definitively characterize their role.

Finally, we acknowledge that treating individual cells as independent observations in differential expression analysis can inflate statistical significance. To address this, we have performed pseudobulk analysis as the primary approach for differential expression, with counts aggregated per cell type per donor and tested using DESeq2 with donor as the biological replicate(**Figure 6h**). Single-cell-level DEG analysis was performed as a sensitivity analysis and is presented in **Supplementary Materials** (**Supplementary Figure S8d**).

### Clinical perspectives

Advanced age, male sex, dysphagia, and V-neck rash have been recognized as significant risk factors for malignancy in DM. Conversely, juvenile onset, ILD, anti-TIF1-β antibodies, and joint involvement are associated with a decreased risk of malignancy (17, 18). Aging therefore plays a critical role in CAM, as it leads to a gradual decline in immune system function, which in turn increases the incidence of diseases such as cancer and infections (64, 65). Our findings indicate that DM patients at high risk of malignancy exhibit a modest increase in Treg cell proportions, reflecting a distinct pattern of peripheral immune dysregulation. Although low-dose IL-2 therapy has demonstrated efficacy in other autoimmune diseases (e.g., SLE) (66), it should be used with caution in DM patients, particularly those positive for anti-TIF1γ antibodies. Moreover, the cancer subtypes associated with DM occur predominantly in the ovary, lung, breast, pancreas, stomach, colorectum, and nasopharynx. Whether these organs or tissues possess mechanisms for recruiting Treg cells remains unknown. Therefore, longitudinal cancer risk assessment and follow-up tracing are required to determine which patients will develop tumors in the coming years and what the tumor types will be.

## Materials and methods

### Study design

This study investigated the PBMC landscape in anti-TIF1γ-positive DM patients, using PBMCs from healthy donors as controls. Peripheral blood was collected from patients meeting the 2020 ENMC-DM classification criteria for DM (7). Patients were positive for anti-TIF1γ at the time of DM diagnosis and had no cancer before or for at least one year after diagnosis. Whole-body CT was performed to screen for cancer after serum anti-TIF1γ positivity was confirmed and the patients provided consent for cancer screening. All blood samples in the disease groups were obtained prior to treatment initiation. The study was approved by the Ethics Review Committee of The Second Affiliated Hospital of Xi’an Jiaotong University (Approval No. 2025146) and conducted in accordance with the Declaration of Helsinki. Written informed consent was obtained from all participants, and the study complies with all applicable ethical regulations for human research.

### Sample preparation

All dermatomyositis patients were diagnosed according to standard clinical and biochemical criteria, including active rash and/or evidence of muscle inflammation [manifested by decreased muscle strength, elevated creatine kinase/lactate dehydrogenase (CK/LDH) levels, and/or MRI findings consistent with myositis] (3). Whole blood samples were collected from both healthy volunteers and anti-TIF1γ-positive DM patients. PBMCs were isolated via density gradient centrifugation and cryopreserved in a solution of 90% fetal bovine serum (FBS) and 10% dimethyl sulfoxide (DMSO). Following controlled rate freezing, samples were processed by Shanghai OE Biotech Co., Ltd. (Shanghai, China) for erythrocyte depletion, quality control assessment, and subsequent sequencing. Detailed reagent information is provided in **Supplementary Table S4**.

### Droplet-based scRNA-seq

Single-cell RNA sequencing libraries were prepared using the Chromium Next GEM Single Cell 3’ Reagent v3.1 kit (10× Genomics) following the manufacturer’s protocol. Single-cell suspensions were loaded onto a Chromium Single-Cell Controller (10× Genomics) to generate single-cell gel bead-in-emulsions (GEMs). Barcoded full-length cDNA was synthesized through reverse transcription, followed by emulsion disruption using recovery reagent. cDNA purification was performed using MyOne Silane Beads (Thermo Fisher Scientific), with subsequent PCR amplification. The amplified cDNA underwent fragmentation, end repair, A-tailing, and adapter ligation. Final libraries were amplified and sequenced on an Illumina NovaSeq platform, generating 150 bp paired-end reads. The raw sequence data reported in this paper have been deposited in the Genome Sequence Archive (Genomics, Proteomics & Bioinformatics 2021) in National Genomics Data Center (Nucleic Acids Res 2022), China National Center for Bioinformation/Beijing Institute of Genomics, Chinese Academy of Sciences (GSA-Human: HRA012299) that are publicly accessible at https://ngdc.cncb.ac.cn/gsa-human (67, 68).

### Processing and quality control of the raw data

Raw sequencing data were processed using Cell Ranger software (v9.0.1; 10× Genomics) to perform demultiplexing, barcode assignment, transcriptome alignment (GRCh38), and read downsampling for cross-sample normalization. This pipeline generated a raw Unique Molecular Identifier (UMI) count matrix, which was subsequently imported into Seurat (v5.1.0) for downstream analysis. Previously derived data from anti-TIF1γ-negative DM groups (including anti-MDA5-positive DM and anti-synthetase syndrome) were also included as controls (13). We applied quality control thresholds to exclude low-quality cells and potential doublets. Cells were sequentially filtered based on UMI counts, the number of detected genes (nFeature_RNA), and the percentage of mitochondrial reads. Specifically, we retained cells with UMI counts between the 5th and 99.5th percentiles and gene counts between the 2nd and 99.5th percentiles. Cells with more than 20% mitochondrial transcripts were also excluded (**Supplementary Figure S1**).

### Quantification of single-cell gene expression and cell type determination

Following quality control, SCTransform was utilized to normalize the data while regressing the detection rate of mitochondrial gene expression. Data integration was performed using harmony (69), resulting in a batch-corrected matrix of 3,000 features via the IntegrateData function. Principal component analysis (PCA) was conducted on these selected genes using the RunPCA function, with the resulting principal components projected across all genes. For cell clustering, we performed graph-based clustering (FindClusters) on the gene expression profiles. Dimensionality reduction was achieved through t-Distributed Stochastic Neighbor Embedding (t-SNE) using Seurat’s RunTSNE function, with two-dimensional visualizations generated by ggplot and DimPlot. Gene expression distributions were visualized using violin plots (VlnPlot). Due to the dataset size, individual data points were omitted from the violin plots to maintain a clear visualization of the overall distributions.

### Single-cell regulatory network inference and clustering analysis

We conducted SCENIC analysis using pySCENIC (version 0.9.18). The correlation coefficients between regulators/TFs and their targets were calculated using the entire expression matrix to generate multiple modules and the positively correlated and most stable modules were retained for further analysis. The co-expression modules were pruned by cis-regulatory motif analysis using RcisTarget to exclude the indirect targets. Direct target genes across all modules sharing the same TF were combined into a single resulting regulon. The activity of the predicted regulon was quantified using the area under the recovery curve.

### Gene set variation analysis

We conducted GSVA utilizing hallmark pathway gene signatures from the Molecular Signatures Database (MSigDB), accessed via the GSEABase package (v1.64.0). To enhance specificity, we refined each pathway-associated gene set by retaining only unique genes and excluding those that were shared among multiple pathways. Pathway activity scores for individual cells were calculated using the default GSVA parameters, and the results were then converted to z-scores for normalization.

### IFN score, Fc gamma R-mediated phagocytosis score, natural killer cell mediated cytotoxicity score, cytotoxicity, Treg suppressive capacity and T cell receptor signaling pathway score

Scores were calculated using the AddModuleScore function in the Seurat package. IFN score was calculated according to published literature (11). The reference datasets of Fc gamma R-mediated phagocytosis (hsa04666), natural killer cell mediated cytotoxicity (hsa04650) and T cell receptor signaling pathway (hsa04660) used for calculating these scores are provided in **Supplementary Table S5**.

### Cell-to-cell communications

CellChat (v1.6.1) was used to analyze cell-cell communication networks and infer interactions based on established ligand-receptor pairs (40, 70). Following the standard workflow of the package, we: (1) loaded normalized transcript counts, (2) identified overexpressed genes and interactions using the functions identifyOverExpressedGenes and identifyOverExpressedInteractions, and (3) projected the data with projectData. For network inference, we incorporated predefined human signaling pathways and protein-protein interactions as prior knowledge. The subsequent analysis included computing communication probabilities (ComputeCommunProb), pathway-level communication (ComputeCommunProbPathway), and network aggregation (aggregateNet), all performed with default parameters and fixed random seeds. Finally, we employed netAnalysis_signallingRole to characterize the sender and receiver cell populations within the network.

### Flow cytometric analysis of CD8^+^ T, NK, and B cell proportions in PBMCs

Peripheral blood samples (**Supplementary Table S2**) were collected and stored in EDTA anticoagulant tubes for analysis within 24 hours. The absolute counts of lymphocytes and monocytes were determined using a Complete Blood Count (CBC) performed on a Sysmex Automated Hematology Analyzers (XN-9100). For the quantification of CD8^+^ T cells, 100 μl of whole blood was stained with 10 μl of the tetraCHROME CD45-FITC/CD4-PE/CD8-ECD/CD3-PC5 Antibody Cocktail for 20 minutes. The samples were then incubated with Optilyse C Lysing Solution (Beckman, A11895) for an additional 15 minutes. To quantify NK and B cells, 100 μl of whole blood was stained with 10 μl of the tetraCHROME CD45-FITC/CD56-PE/CD19-ECD/CD3-PC5 Antibody Cocktail for 20 minutes, followed by a 15-minute incubation with Optilyse C Lysing Solution (Beckman, A11895). Subsequently, the cells were analyzed using a Navios flow cytometer, following established templates. The proportions of CD8^+^ T cells, NK cells, and B cells within the total PBMCs were calculated accordingly.

### Flow cytometric analysis of Treg cell proportions in PBMCs

Freshly isolated PBMCs (**Supplementary Table S3**) were washed twice with 2 ml of Flow Cytometry Staining Buffer (Proteintech, PF00018) and then resuspended at a concentration of 1 × 10^7^ cells/ml. For each sample, 100 μl of cell suspension was incubated with 5 μl of PE Mouse Anti-Human CD4 Antibody (STARTER, S-574-16) and 1.25 μl of APC Mouse Anti-Human CD25 Antibody (STARTER, S-R494) at 4 °C for 30 minutes. After washing with Flow Cytometry Staining Buffer, the cells were fixed and permeabilized using the Foxp3/Transcription Factor Staining Buffer Kit (Proteintech, PF00011) according to the manufacturer’s protocol. The cells were then intracellularly stained with 1.25 μl of Alexa Fluor^®^ 488 Mouse Anti-Human FOXP3 Antibody (STARTER, S-2903). Subsequently, the cells were analyzed using a BD Canton II flow cytometer following established templates. The proportions of Treg cells (CD4^+^CD25^+^FOXP3^+^) within total PBMCs were calculated accordingly.

### Immunohistochemistry

Skin biopsies obtained from the patients were immediately fixed in 10% neutral-buffered formalin to preserve tissue morphology and antigenicity. Following fixation, the samples were routinely processed, embedded in paraffin wax, and sectioned at a thickness of 5 μm using a microtome. IHC staining for FOXP3 was performed on the paraffin-embedded tissue sections using the IHCeasy^®^ FOXP3 Ready-To-Use IHC Kit (KHC2510, Proteintech), strictly following the manufacturer’s protocol. This marker was employed to identify Treg cells within the skin infiltrate. In addition, H&E staining was carried out on adjacent tissue sections according to standard histological protocols to evaluate general tissue architecture and inflammatory cell infiltration.

### Statistics

All statistical analyses were conducted using R (v4.3.3) and GraphPad Prism 9. Bar plots display mean ± SD unless otherwise specified. Heatmaps were created using the ComplexHeatmap package. For group comparisons, unpaired two-tailed *t*-tests were used for two-group analyses. Differentially expressed genes (DEGs) were identified either using the FindMarkers function in Seurat followed by Wilcoxon rank-sum tests or DESeq2, with donor as the biological replicate. Linear models were generated when multiple parameters were taken into account. A *p*-value threshold of <0.05 was considered statistically significant.

## Data Availability

The datasets presented in this study can be found in online repositories. The names of the repository/repositories and accession number(s) can be found in the article/**Supplementary Material**.
