# Supplementary material for: Dysregulation of the cytotoxic-humoral axis and expansion of regulatory T cells in anti-TIF1γ- positive dermatomyositis
Source: Front Immunol. 2026 Sep 18;17:1921818. doi: 10.3389/fimmu.2026.1921818 (PMC13631053; doi:10.3389/fimmu.2026.1921818)
Supplement: Supplementary file 1 [file DataSheet1.pdf]

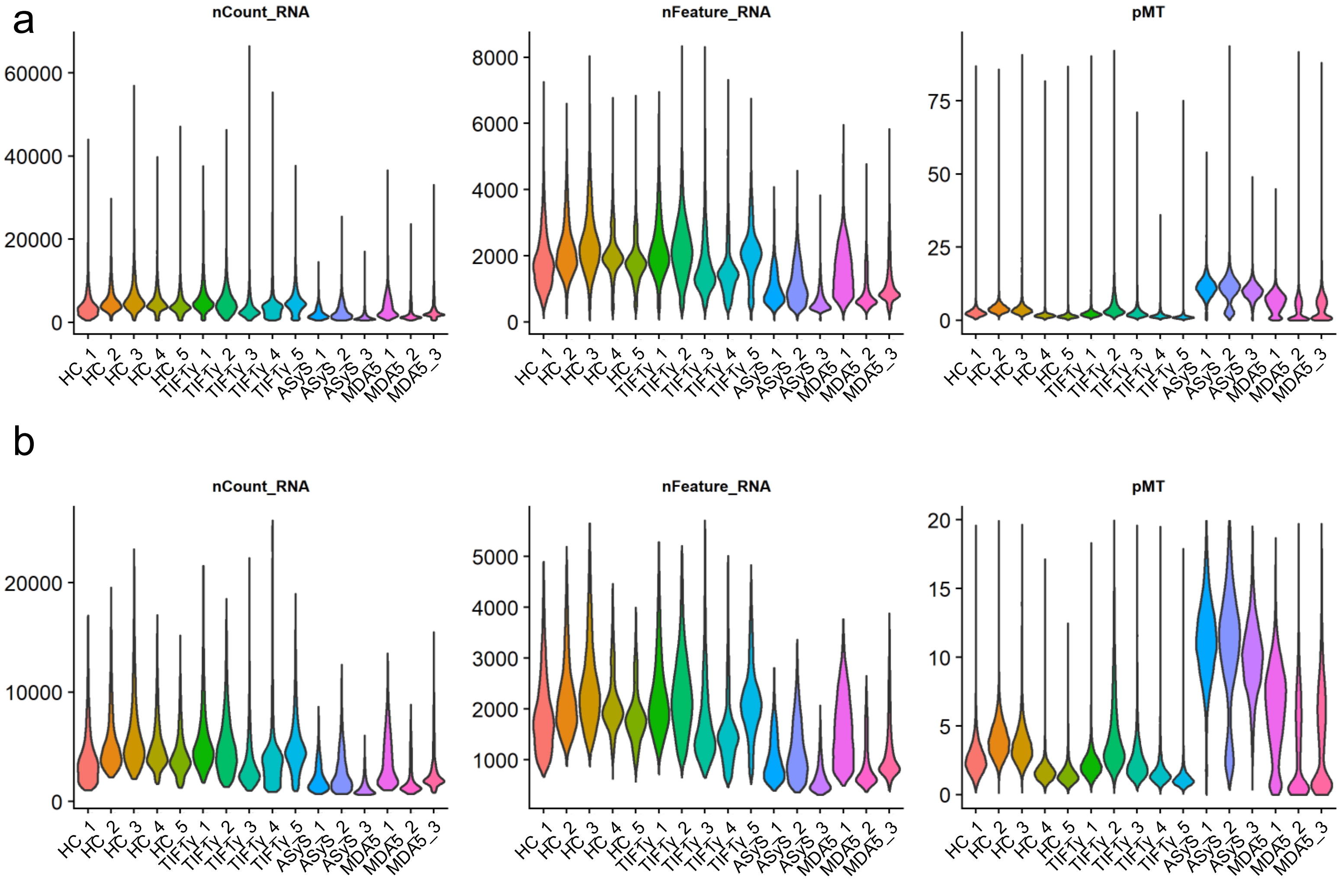

Figure S1. Quality control of scRNA-seq data. Violin plots of UMIs (left), gene numbers (middle) and percentage of mitochondrial genes (right) of each samples [HC (HC_1-5), TIF1γ (TIF1γ_1-5), ASyS (ASyS_1-3), and MDA5 (MDA5_1-3] before (a) and after (b) quality control.

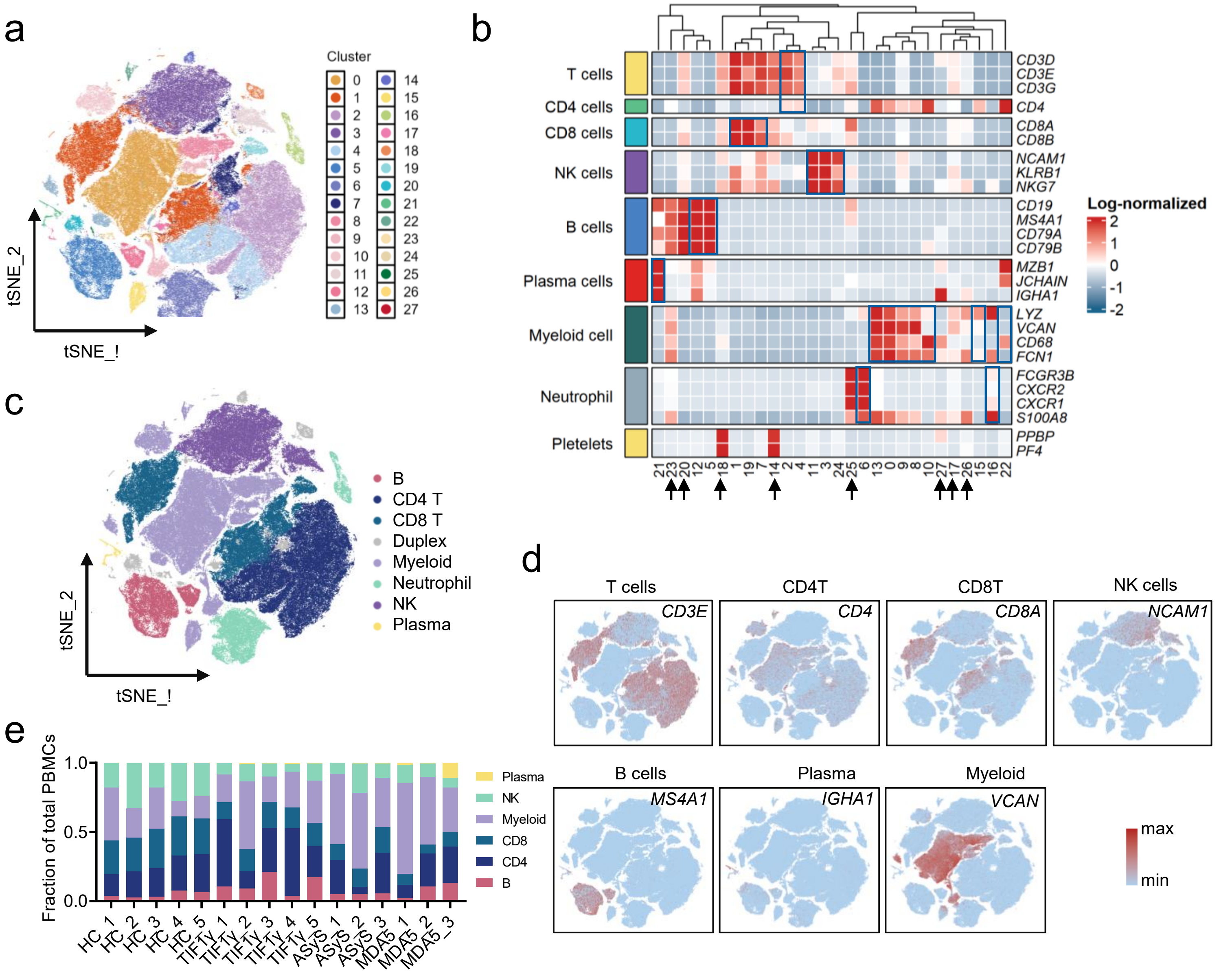

Figure S2. Cell typing of PBMCs in anti-TIF1γ-positive DM relative to HC through scRNA-seq. a) tSNE plot of the 28 clusters with each cell color-coded for the associated cluster. **b**) Heatmap illustrating the classical markers of different cell types. Matched clusters are highlighted in blue. Arrows are used to identify clusters that are duplexes. **c**) tSNE plot of the 185,619 cells profiled with each cell color-coded for cell types. Duplex are also shown. **d**) tSNE plot of all the cells with each cell color-coded for the expression of a marker gene for the individual cell subtypes. **e**) Proportion plots of major cell types in HC (HC_1-5), TIF1γ (TIF1γ_1-5), ASyS (ASyS_1-3), and MDA5 (MDA5_1-3) PBMCs derived from different individuals.

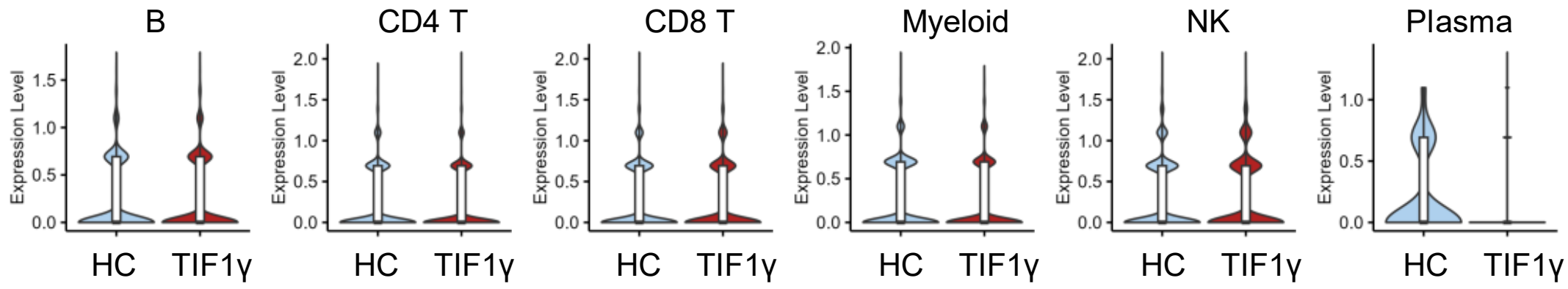

Figure S3. Violin plots of the expression distribution of TRIM33 in the two groups (HC and anti-TIF1γ-positive DM) of B, CD4 T, CD8 T, myeloid, NK and Plasma cells.

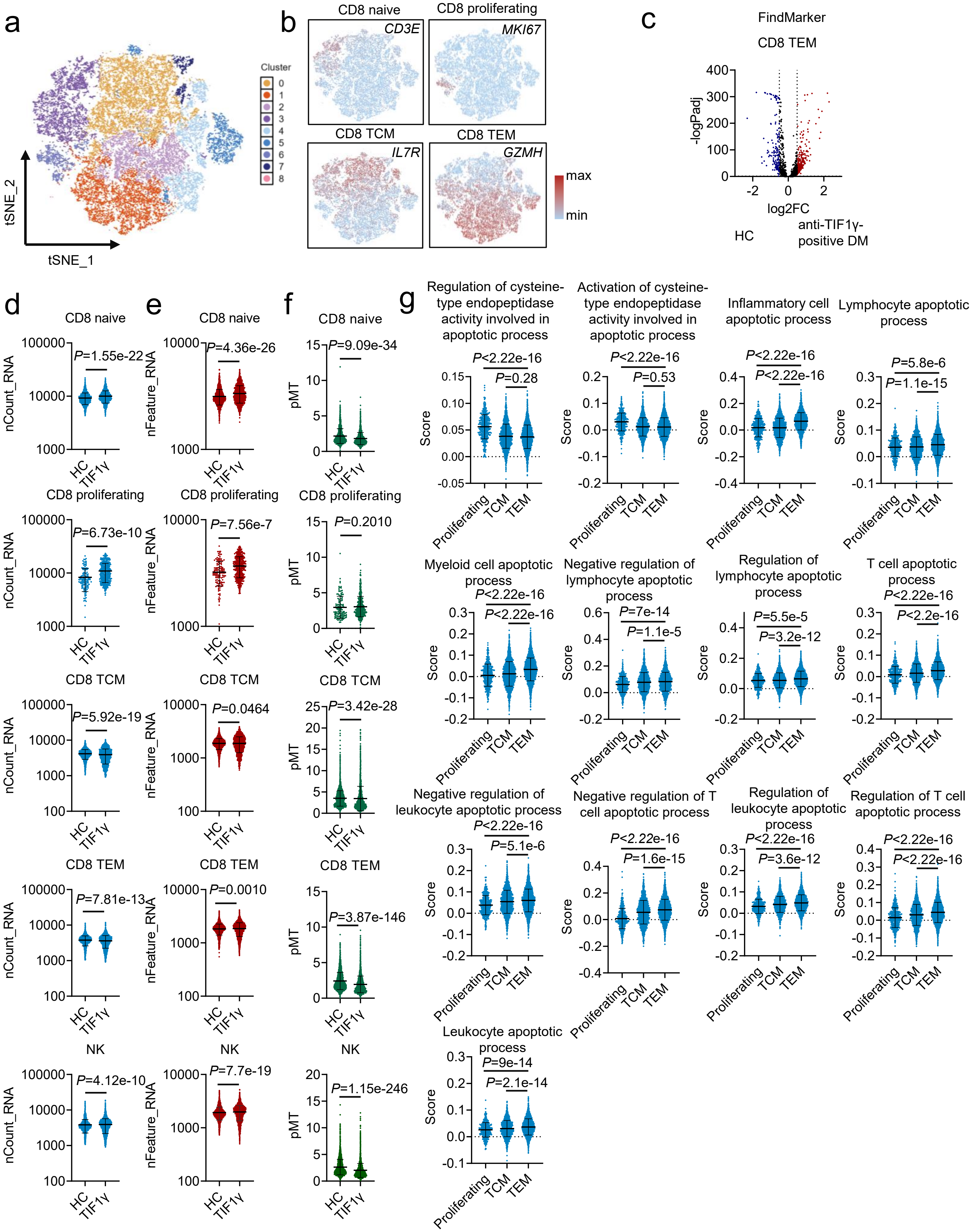

a
b
c
d
e
f
g
CD8 naive
CD8 proliferating
CD8 TCM
CD8 TEM
FindMarker
tSNE_1
tSNE_2
Cluster
HC
anti-TIF1γ-positive DM
log2FC
-logPadj
nCount_RNA
nFeature_RNA
pMT
Score
NK

Figure S4. Reduced cytotoxic cell frequency with enhanced IFN signature in anti-TIF1γ-positive DM. **a**) tSNE plot of the $CD8^+$ T cells in 9 clusters with each cell color-coded for the associated cluster. **b**) tSNE plot of all the cells with each cell color-coded for the expression of a marker gene for the individual $CD8^+$ T cell subtype. **c**) Volcano plot of the differential expression genes of CD8 TEM in anti-TIF1γ-positive DM versus HC. **d-f**) Scatter plots of UMIs (d), gene numbers (e) and percentage of mitochondrial genes (f) in the two groups of CD8 T subtypes and NK cells. **g**) The scores of the 13 cell death-associated pathways (related to figure 3e) in proliferating $CD8^+$ T cells, $CD8^+$ TCM, and $CD8^+$ TEM in TIF1γ group. Data (d-g) are mean±SD, Wilcoxon rank-sum test..

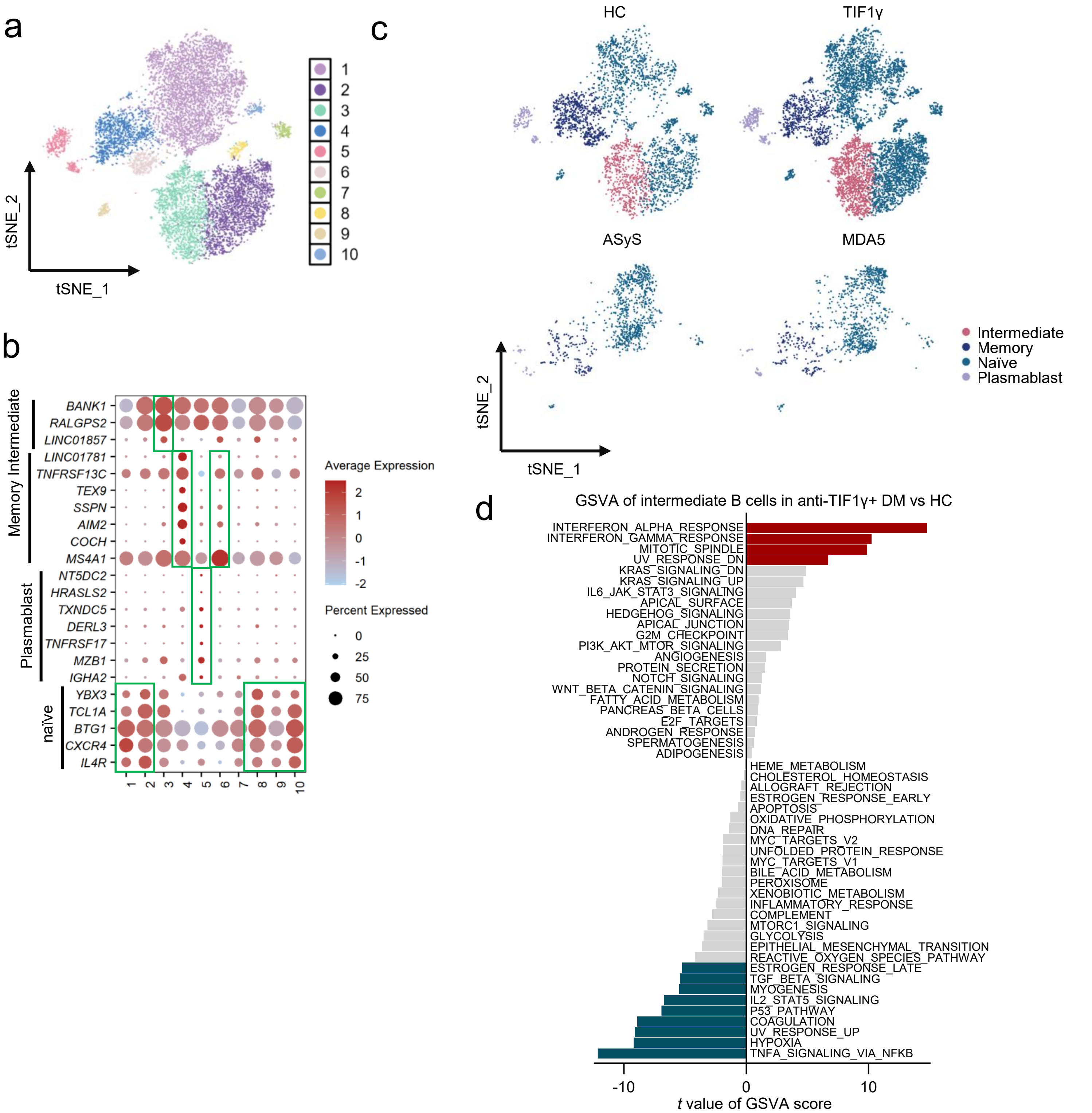

Figure S5 Increased naïve and intermediate B cell frequency in peripheral blood of anti-TIF1γ-positive DM patients. **a**) tSNE plot of the B cells in 10 clusters with each cell color-coded for the associated cluster. **b**) Dot plots of cell type marker genes. Expression values were normalized and scaled to the averages. **c**) tSNE plot of B cells subtypes split by groups (HC, TIF1γ, ASyS and MDA5), color-coded by B cells subtypes. **d**) Differential expression of hallmark pathway gene signatures scored per cell using gene set variation analysis (GSVA) of intermediated B cells in anti-TIF1γ-positive DM versus HC. The data shown are *t* values from a linear model Box plots of UMIs detected in intermediate B cells.

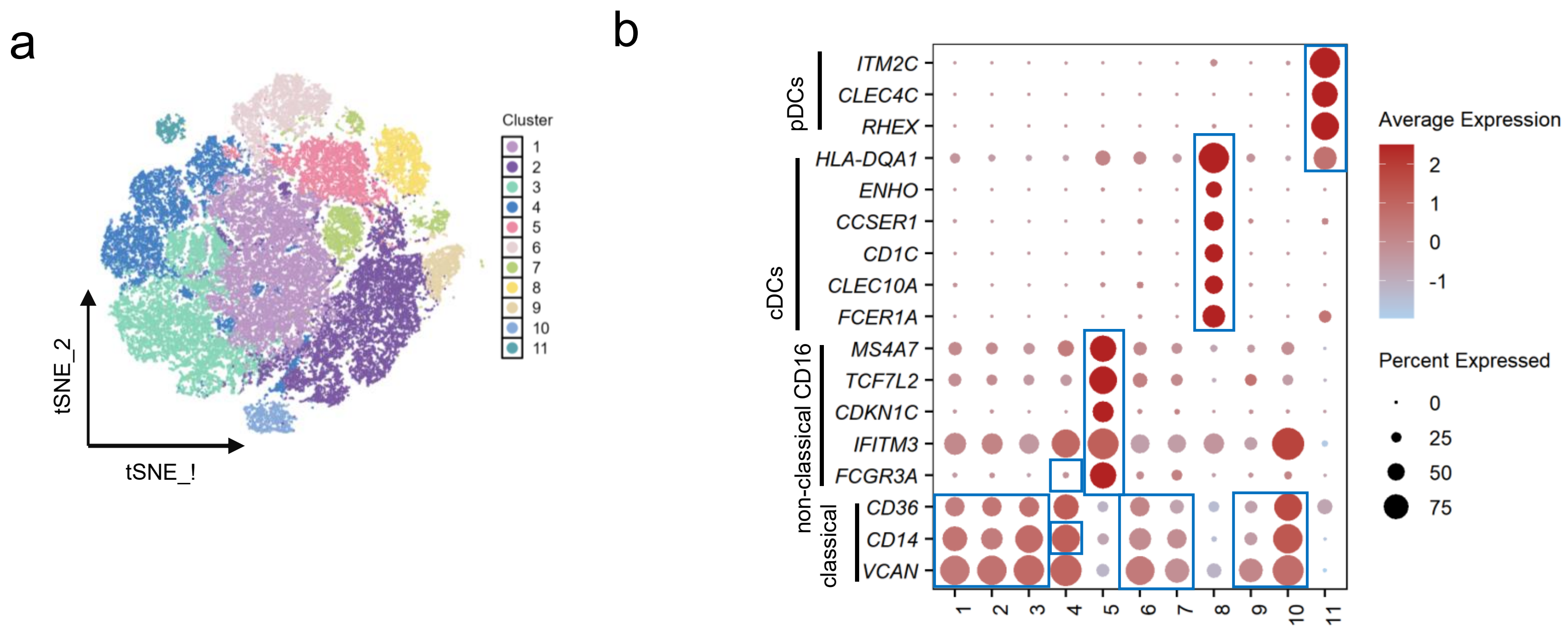

Figure S6 Non-classical monocytes in anti-TIF1γ-positive PBMCs show a reduced proportion and impaired phagocytic capacity. a) tSNE plot of the monocytes and DCs in 11 clusters with each cell color-coded for the associated cluster. **b**) Dot plots of cell type marker genes. Expression values were normalized and scaled to the averages.

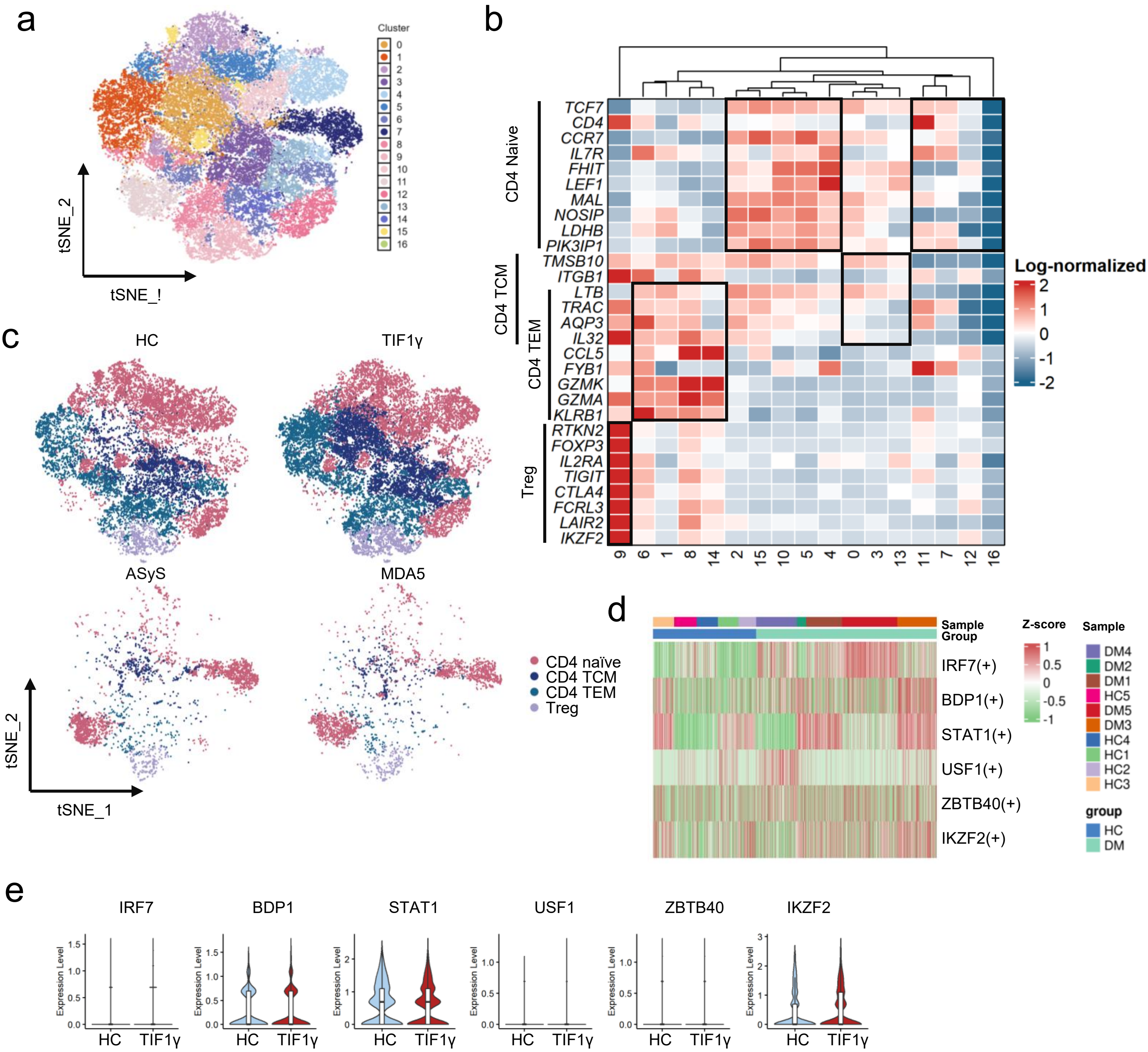

Figure S7 Increased frequency of regulatory T cells in anti-TIF1γ-positive DM. a) tSNE plot of CD4⁺ T cells cluster with each cell color-coded for the associated clusters. **b**) Heatmap illustrating the classical markers of different CD4⁺ T subtypes. Matched clusters are highlighted in black. **c**) tSNE plot of CD4⁺ T cells subtypes split by groups (HC, TIF1γ, ASyS and MDA5), color-coded by CD4⁺ T cells subtypes. **d**) Heatmap displaying the area under the curve (AUC) scores for transcription factor binding motifs in anti-TIF1γ-positive DM Treg cells compared to HC Treg cells, as determined by SCENIC. The data highlights the top six transcription factors with the highest z-scores in anti-TIF1γ-positive DM Treg cells. **e**) Violin plots of the expression distribution of IRF7, BDP1, STAT1, USF1, ZBTB40 and IKZF2 in the two groups of Treg cells.

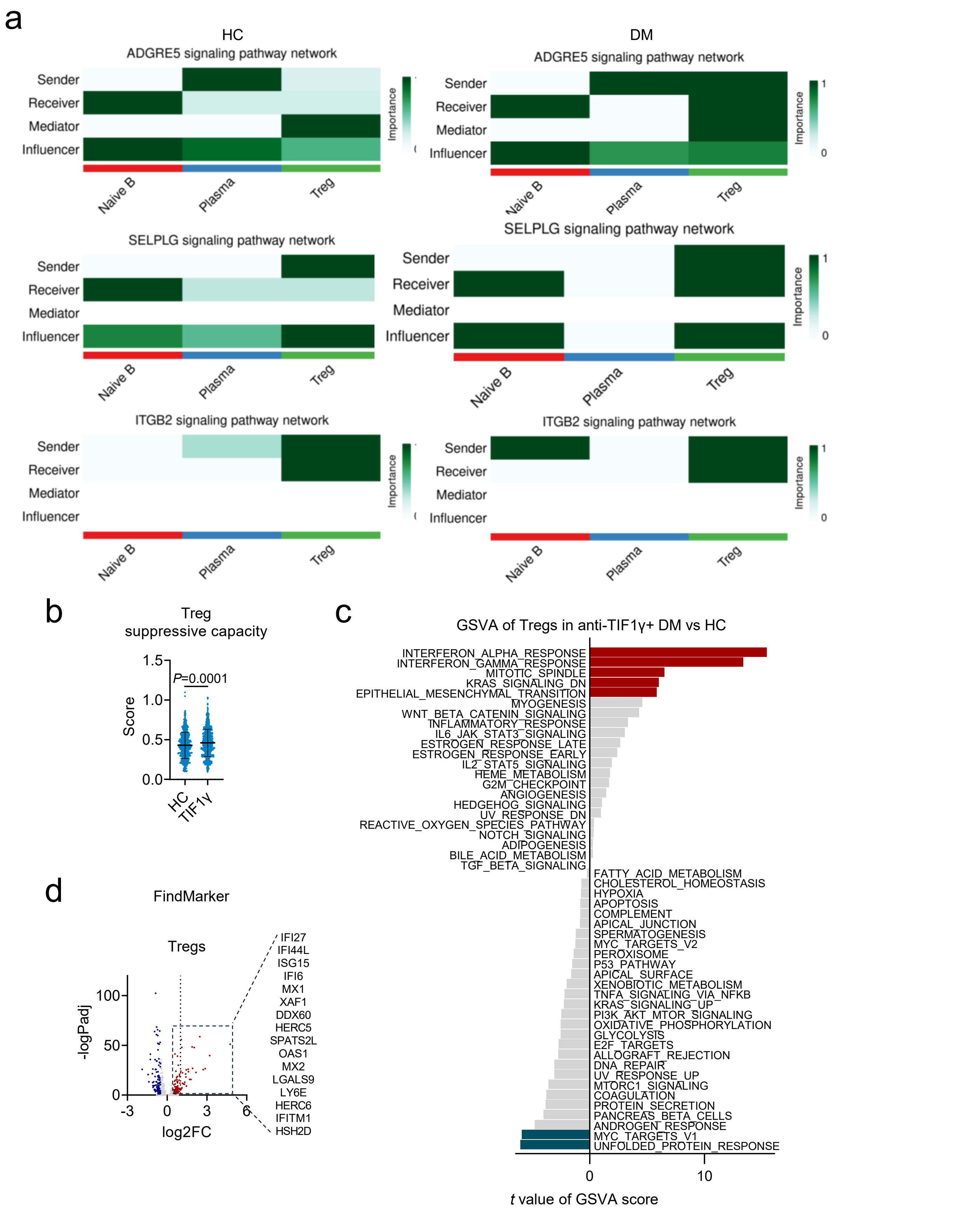

Figure S8 Increased frequency of regulatory T cells in anti-TIF1γ-positive DM. a) Heatmaps showing relative importance based on the computed four network centrality measures (signalling sources, targets, mediators and influencers) of the Adhesion G Protein-Coupled Receptor E5 (ADGRE5, top), Selectin P Ligand (SELPLG, middle) and Integrin Subunit Beta 2 (ITGB2, bottom) signaling pathway. **b**) The scores of the Treg suppressive capacity of Tregs in TIF1γ and HC groups. **c**) Differential expression of hallmark pathway gene signatures scored per cell using gene set variation analysis (GSVA) of Treg cells in anti-TIF1γ-positive DM versus HC. The data shown are *t* values from a linear model Box plots of UMIs detected in Treg cells. **d**) Volcano plot of the differential expression genes of Treg cells in anti-TIF1γ-positive DM versus HC. IFN-inducible genes are also indicated. Data b are mean±SD, Wilcoxon rank-sum test.
