# Supplementary material for: Dysregulation of the cytotoxic-humoral axis and expansion of regulatory T cells in anti-TIF1γ- positive dermatomyositis
Source: Front Immunol. 2026 Sep 18;17:1921818. doi: 10.3389/fimmu.2026.1921818 (PMC13631053; doi:10.3389/fimmu.2026.1921818)
Supplement: Supplementary file 2 [file Table1.docx]

**Supplemental tables**

**Table S1 Summary of clinicopathological characteristics of the participants for scRNA-seq**

| **ID** | **Age** | **Gender** | **anti-TIF1-γ** | **CK (40-200) U/L** | **ALT (7-40) IU/L** | **AST (13-35) IU/L** | **CRP (<2) mg/L** | **ESR (M: 0-15; F: 0.-20) mm/h** | **ANA titer** | **Monocytes （×10^9^ cells/L)** | **lymphocytes （×10^9^ cells/L)** |
| --- | --- | --- | --- | --- | --- | --- | --- | --- | --- | --- | --- |
| HC_1 | 67 | F | NA | NA | 21 | 28 | NA | NA | NA | 0.39 | 2.02 |
| HC_2 | 70 | M | NA | NA | 22 | 24 | NA | NA | NA | 0.25 | 1.65 |
| HC_3 | 29 | M | NA | NA | 45 | 27 | NA | NA | NA | 0.36 | 2.09 |
| HC_4 | 49 | F | NA | NA | 14 | 19 | NA | NA | NA | 0.17 | 1.34 |
| HC_5 | 53 | F | NA | NA | 12 | 21 | NA | NA | NA | 0.33 | 2.09 |
| TIF1γ_1 | 71 | F | YES | 1399 | 167 | 197 | 6.5 | 8 | 1:320 | 0.39 | 0.41 |
| TIF1γ_2 | 67 | M | YES | 95 | 12 | 19 | 3.58 | 8 | 1:100 | 0.58 | 0.72 |
| TIF1γ_3 | 55 | F | YES | 106 | 66 | 51 | 0.5 | 10 | 1:100 | 0.43 | 1.15 |
| TIF1γ_4 | 47 | M | YES | 92 | 23 | 25 | 22.81 | 51 | 1:100 | 0.5 | 0.61 |
| TIF1γ_5 | 78 | F | YES | 97 | 14 | 16 | 0.5 | 6 | 1:100 | 0.22 | 0.28 |

Note: F: female; M: male; NA, not available; CK, creatine kinase; ALT, Alanine aminotransferase; AST, aspartate aminotransferase; CRP, C-reactive protein; ESR, erythrocyte sedimentation rate; ANA, antinuclear antibody.

**Table S2 Summary of clinicopathological characteristics of the participants for CD8^+^ T, CD4^+^ T,NK and B cell quantification**

| **Characteristics** | **HC (n=40)** | **anti‑TIF1γ‑positive DM (n=21)** |
| --- | --- | --- |
| Age (Mean ± SD) | 44.6 ± 6.2 | 49.6 ± 16.6 |
| Gender |  |  |
| Male | 14 (35.0%) | 5 (23.8%) |
| Female | 26 (65.0%) | 16 (76.2%) |
| Lymphocyte counts [×10^9^/L, (Mean ± SD)] | 1.93 ± 0.42 | 1.08 ± 0.49 |
| Monocyte counts [×10^9^/L, (Mean ± SD)] | 0.40 ± 0.12 | 0.35 ± 0.15 |

**Table S3 Summary of clinicopathological characteristics of the participants for CD4^+^ T and Treg cell quantification**

| **Characteristics** | **HC (n=14)** | **anti‑TIF1γ‑positive DM (n=6)** |
| --- | --- | --- |
| Age (Mean ± SD) | 47.6 ± 11.3 | 60.6 ± 14.2 |
| Gender |  |  |
| Male | 10 (71.4%) | 2 (33.3%) |
| Female | 4 (28.6%) | 4 (67.7%) |
| Lymphocyte counts [×10^9^/L, (Mean ± SD)] | 2.04 ± 0.59 | 0.92 ± 0.41 |
| Monocyte counts [×10^9^/L, (Mean ± SD)] | 0.44 ± 0.14 | 0.57 ± 0.35 |

**Table S4. List of regents**

| **Reagent** | **Catalogue No.** | **Company** |
| --- | --- | --- |
| Human Peripheral Blood Lymphocyte Separation Medium | C0025-200ml | Beyotime (Shanghai, China) |
| Hanks' Balanced Salt Solution | C0218 | Beyotime (Shanghai, China) |
| tetraCHROME CD45-FITC/CD4-PE/CD8-ECD/CD3-PC5 Antibody Cocktail | 6607013 | Beckman (California, USA) |
| tetraCHROME CD45-FITC/CD56-PE/CD19-ECD/CD3-PC5 Antibody Cocktail | 6607073 | Beckman (California, USA) |
| Optilyse C Lysing Solution | A11895 | Beckman (California, USA) |
| PE Mouse Anti-Human CD4 Antibody | S-574-16 | Starter (Hangzhou, China) |
| APC Mouse Anti-Human CD25 Antibody | S-R494 | Starter (Hangzhou, China) |
| Alexa Fluor® 488 Mouse Anti-Human FOXP3 Antibody | S-2903 | Starter (Hangzhou, China) |
| Foxp3 Transcription Factor Staining Buffer Kit | PF00011 | Proteintech (Wuhan, China) |
| Flow Cytometry Staining Buffe | PF00018 | Proteintech (Wuhan, China) |
| Hematoxylin-Eosin (HE) Stain Kit | G1120 | Solarbio (Beijing, China) |

**Table S5** **The reference genes to calculate scores.**

| **Pathways** | **Genes** |
| --- | --- |
| **IFN** | IFI6, UBE2L6, IFITM3, IFITM2, MT2A, SP100, OAS1, BST2, XAF1, IFI35, ISG15, USP18, OASL, OAS2, OAS3, IFIT5, RNASEL, RSAD2, IFIT3, IFIT1, DDX58, IFIT2, EIF4E2, STAT2, ADAR, PML, PTPN6, GBP2, UBA7, IF130, ISG20, B2M, SUMO1, IRF9, NUP37, PIN1, IP6K2, NUP54, TPR, NUP88, IRF3, NUP188, NUP210, JAK1, PSMB8, PTPN1, NUP107, AAAS, IFNG, IFITM1, NUP155, NEDD4, NUP153, NUP62, KPNB1, IFNGR2, PTAFR, TYK2, JAK2, SAMHD1, KPNA1, IFNAR1, PIAS1, NUP214, NUP50, SEC13, CIITA, IFI27, NUP43, NUP93, NUP160, FLNB, FLNA, NUP205, PDE12, NUP35, NUP85, RAE1, IFNGR1, PPM1B, PTPN11, RANBP2, NUP133, ABCE1, NDC1, EGR1 |
| **Fc gamma R-mediated phagocytosis** | AKT3, PLA2G4B, DNM1L, ARPC5, ARPC4, ARPC3, ARPC1B, ARPC2, WASF2, VAV3, ARPC1A, CFL1, CFL2, WASF3, PLA2G4E,  CRK, CRKL, DNM1, DNM2, DOCK2, PIKFYVE, AKT1, AKT2, FCGR1A, FCGR2A, FCGR2B, FCGR3A, PIP5K1C, PIK3R5, PLA2G4F, DNM3, LAT, AMPH, PLA2G4D, GSN, HCK, INPP5D, ARF6, LIMK1, LIMK2, LYN, MARCKS, MYO10, PAK1, ASAP1,  PIK3CA, PIK3CB, PIK3CD, PIK3CG, PIK3R1, PIK3R2, PLA2G4A, PLCG1, PLCG2, PLD1, PLD2, ASAP3, PRKCA, PRKCB, PRKCD,  PRKCE, PRKCG, MAPK1, MAPK3, MAP2K1, SPHK2, PTPRC, RAC1, RAC2, RAF1, RPS6KB1, RPS6KB2, MARCKSL1, NCF1, SYK, VASP, VAV1, VAV2, WAS, ARPC5L, PIP5K1A, PIP5K1B, PIP4K2B, PLA2G6, PIK3R3, SCIN, PLPP1, PLPP2, PLPP3, ASAP2,  SPHK1, WASF1, WASL, FCGR2C, GAB2, CDC42 |
| **Natural killer cell mediated cytotoxicity** | KIR2DS2, MICA, KLRC4-KLRK1, IGH, VAV3, HCST, P3R3URF-PIK3R3, SH2D1B, FCGR3B, RAET1E, CSF2, RAET1L, PTK2B, FCER1G, FCGR3A, FCGR3B, KLRK1, FYN, SHC2, NCR3, LAT, GRB2, GZMB, HLA-A, HLA-B, HLA-C, HLA-E, HLA-G, HRAS, ICAM1, ICAM2, IFNA1, IFNA2, IFNA4, IFNA5, IFNA6, IFNA7, IFNA8, IFNA10, IFNA13, IFNA14, IFNA16, IFNA17, IFNA21, IFNAR1, IFNAR2, IFNB1, IFNG, IFNGR1, IFNGR2, RAET1G, FAS, FASLG, ITGAL, ITGB2, ARAF, KIR2DL1, KIR2DL2, KIR2DL3,  KIR2DL4, KIR2DS1, KIR2DS3, KIR2DS4, KIR2DS5, KIR3DL1, KIR3DL2, KLRC1, KLRC2, KLRC3, KLRD1, KRAS, LCK, LCP2,  SHC4, SH2D1A, MICB, NFATC1, NFATC2, NRAS, PAK1, CD244, PIK3CA, PIK3CB, PIK3CD, PIK3R1, PIK3R2, PLCG1, SHC3, PLCG2, PPP3CA, PPP3CB, PPP3CC, PPP3R1, PPP3R2, PRF1, PRKCA, PRKCB, PRKCG, MAPK1, MAPK3, MAP2K1, MAP2K2, KIR2DL5A, PTPN6, PTPN11, RAC1, RAC2, RAC3, RAF1, BID, SH3BP2, SHC1, SOS1, SOS2, BRAF, SYK, TNF, TYROBP, VAV1, VAV2, LAT2, ZAP70, ULBP3, ULBP2, ULBP1, CASP3, PIK3R3, TNFSF10, TNFRSF10B, TNFRSF10A, CD247, NCR2, NCR1, CD48 |
| **T cell receptor signaling pathway** | AKT3, RASGRP1, CDK4, PAK4, VAV3, BUB1B-PAK6, MALT1, P3R3URF-PIK3R3, CHUK, MAP3K8, MAPK14, CSF2, CTLA4, DLG1, AKT1, AKT2, FOS, FYN, LAT, PPP2R3B, GRB2, GSK3B, ICOS, HRAS, IFNG, IKBKB, IL2, IL4, IL5, IL10, ITK, JUN, KRAS, RHOA, LCK, LCP2, NCK1, NFATC1, NFATC2, NFATC3, NFKB1, NFKBIA, NFKBIB, NFKBIE, NRAS, PAK1, PAK2, PAK3, PDCD1, PDPK1, PIK3CA, PIK3CB, PIK3CD, PIK3R1, PIK3R2, PLCG1, PPP2R3C, PPP2CA, PPP2CB, PPP2R1A, PPP2R1B, PPP2R2A, PPP2R2B, PPP2R2C, PPP2R3A, PPP2R5A, PPP2R5B, PPP2R5C, PPP2R5D, PPP2R5E, PPP3CA, PPP3CB, PPP3CC, PPP3R1, PPP3R2, PPP2R2D, PRKCQ, MAPK1, MAPK3, MAPK8, MAPK11, MAPK9, MAPK10, MAPK13, MAP2K1, MAP2K2, MAP2K7, PAK6, PAK5, PTPN6, PTPN11, PTPRC, RAF1, RELA, MAPK12, SOS1, SOS2, MAP3K7, TEC, TNF, VAV1, VAV2, ZAP70, NCK2, CARD11, PIK3R3, IKBKG, CBLB, BCL10, MAP3K14, CD3D, CD3E, CD3G, CD247, CD4,CD8A, CD8B,C D8B2, CD28, GRAP2, CD40LG, CDC42, |
| **Cytotoxicity** | CST7, GZMA, GZMB, IFNG, NKG7, PRF1 |
| **Treg suppressive capacity** | CIITA, HLA‑DRA, HLA‑DRB1, HLA‑DRB3, HLA‑DRB4, HLA‑DRB5, HLA‑DQA1, HLA‑DQA2, HLA‑DQB1, HLA‑DQB2, HLA‑DQB3, HLA‑DPA1, HLA‑DPB1, CCR7, ENTPD1 |
